# Systematics of *Phyllocnistis* leaf-mining moths (Lepidoptera, Gracillariidae) feeding on dogwood (*Cornus* spp.) in Northeast Asia, with the description of three new species

**DOI:** 10.3897/zookeys.736.20739

**Published:** 2018-02-08

**Authors:** Natalia Kirichenko, Paolo Triberti, Shigeki Kobayashi, Toshiya Hirowatari, Camiel Doorenweerd, Issei Ohshima, Guo-Hua Huang, Min Wang, Emmanuelle Magnoux, Carlos Lopez-Vaamonde

**Affiliations:** 1 Sukachev Institute of Forest SB RAS, Akademgorodok 50/28, 660036, Krasnoyarsk, Russia; 2 Siberian Federal University, 79 Svobodny pr., 660041, Krasnoyarsk, Russia; 3 INRA, UR0633 Zoologie Forestière, F-45075 Orléans, France; 4 Museo Civico di Storia Naturale, Lungadige Porta Vittoria 9, I37129, Verona, Italy; 5 Entomological Laboratory, Graduate School of Life and Environmental Science, Osaka Prefecture University, Sakai, Osaka, 599-8531, Japan; 6 Entomological Laboratory, Faculty of Agriculture, Kyushu University, 6-10-1 Hakozaki, 812-8581, Fukuoka, Japan; 7 Department of Plant and Environmental Protection Sciences, University of Hawaii, 3050 Maile Way, 96822, Honolulu, United States of America; 8 Naturalis Biodiversity Centre, PO Box 9557, NL-2300 RA Leiden, The Netherlands; 9 Department of Life and Environmental Sciences, Kyoto Prefectural University, 606-8522, Kyoto, Japan; 10 Hunan Provincial Key Laboratory for Biology and Control of Plant Diseases and Insect Pests, Hunan Agricultural University, Changsha 410128, Hunan, China; 11 Department of Entomology, South China Agricultural University, Guangzhou 510642, Guangdong, China; 12 Institut de Recherche sur la Biologie de l’Insecte, CNRS UMR 7261, Université François-Rabelais de Tours, UFR Sciences et Techniques, 37200 Tours, France

**Keywords:** *Phyllocnistis*, new species, *Cornus*, Russia, Japan, China, DNA barcoding, nuclear genes, *Wolbachia*

## Abstract

During an ongoing DNA-barcoding campaign of the leaf-mining moths that feed on woody plants in Northeast Asia, four lineages of the genus *Phyllocnistis* (Gracillariidae, Phyllocnistinae) were discovered on dogwood (*Cornus* spp): *P. cornella* Ermolaev, 1987 on *C. controversa* Hemsl. (Japan: Hokkaido) and three new species – one feeding on *C. controversa*, *C. florida* L. and *C. macrophylla* Wall. in Japan (Honshu, Shikoku, Kyushu), a second species on *C. macrophylla* in China (Yunnan) and a third on Siberian dogwood *Cornus alba* L. in Russia (Siberia). All these species showed differences in morphology, in the barcode region of the cytochrome c oxidase I gene and in two nuclear genes (histone H3 and 28S ribosomal RNA). No correlation was found between the deep mitochondrial splits observed and the *Wolbachia* infection pattern. Based on both morphological and molecular evidence, the three recently discovered lineages are described here as new species: *P. indistincta* Kobayashi & Triberti, **sp. n.** (Japan), *P. saepta* Kirichenko, Ohshima & Huang, **sp. n**. (China) and *P. verae* Kirichenko, Triberti & Lopez-Vaamonde, **sp. n.** (Russia). In addition, the authors re-describe the adult morphology of *P. cornella*, provide the first record of this species from Japan and highlight the diagnostic characters that allow these *Cornus*-feeding *Phyllocnistis* species to be distinguished.

## Introduction

Leaf-mining micromoths of the family Gracillariidae have been the focus of recent taxonomic studies in particular in the Palaearctic region (Langmaid and Corley 2007, Triberti 2007, Laštůvka et al. 2013, Doorenweerd et al. 2014, Kirichenko et al. 2015, 2016, Huemer et al. 2016). Amongst the 100 plus genera of gracillariids known worldwide, the genus *Phyllocnistis* Zeller, 1848 belongs to the subfamily Phyllocnistinae Herrich-Schäffer, 1857 (Kawahara et al. 2017) and still remains poorly studied (Davis and Miller 1984, Kawahara et al. 2009, Brito et al. 2012). *Phyllocnistis* adults are small, with wingspan generally less than 5 mm and difficult to identify to species level since they show little morphological differentiation in genitalia (De Prins and Kawahara 2009). However, some studies have shown that the morphology of the pupal head and abdomen can provide valuable diagnostic characters (Kawahara et al. 2009, Davis and Wagner 2011, Kobayashi et al. 2011a).

To date, 109 species have been recognised within the genus *Phyllocnistis* (De Prins and De Prins 2017, Brito et al. 2017a, 2017b, Fochezato et al. 2017). Amongst them, 22 new species have been discovered and described in the last decade: 16 species in South America (i.e. Brazil, Costa Rica, Colombia, French Guiana) (Kawahara et al. 2009, Davis and Wagner 2011, Brito et al. 2012, 2017a, 2017b, Fochezato et al. 2017), three in the USA (Davis and Wagner 2011), two in Japan (Kobayashi et al. 2011b, Kobayashi and Hirowatari 2011) and one in Europe (Langmaid and Corley 2007).

*Phyllocnistis* species are found in six biogeographical realms: 36 species are known from the Oriental realm, 28 species from the Neotropics, 18 species from the Palaearctic, 17 species from Australasia, 16 species from the Nearctic and four species from the Afrotropics; five species occur in more than one realm, partly through introductions (De Prins and De Prins 2017). Host plants are known for 74 species (De Prins and De Prins 2017, Brito et al. 2017a, 2017b, Fochezato et al. 2017). Larvae develop on plants from 21 orders and 34 families. Despite the broad host plant range, *Phyllocnistis* species show a high level of host plant specificity, often feeding on a single host plant genus or species.

Until recently, only one species, *P. cornella* Ermolaev, 1987, has been recorded feeding on dogwood *Cornus* (Cornales: Cornaceae). It was described from the Russian Far East, from Kunashir, the southernmost island of the Kuril Islands, on *C. controversa* (Ermolaev 1987). V.P. Ermolaev based his description on the morphology of the male genitalia and forewing pattern, providing a short description of the species.

The Eastern part of Eurasia, with its vast forests and little explored mountain ranges, still holds many new species of Lepidoptera. During an ongoing DNA-barcoding campaign of Gracillariidae leaf-mining moths from Northeast Asia, four divergent *Phyllocnistis* lineages feeding on *Cornus* spp. were detected: two from Japan, one from China and one from Russia. DNA barcoding, nuclear data and morphology of adults confirmed the presence of three new species that are described here: *P. indistincta* Kobayashi & Triberti sp. n. (Japan: Honshu, Shikoku, Kyushu), *P. saepta* Kirichenko, Ohshima & Huang sp. n. (China: Yunnan) and *P. verae* Kirichenko, Triberti & Lopez-Vaamonde, sp. n. (Russia: Siberia). In addition, the authors also re-describe the adult morphology of the closely related East Asian *P. cornella*, highlighting diagnostic characters that help to distinguish *Cornus*-feeding *Phyllocnistis* and provide the first records of *P. cornella* from Japan (Hokkaido). Female genitalia and pupal morphology of *P. cornella* are described here for the first time.

## Materials and methods

In total, 212 specimens have been studied (Suppl. material 1). One hundred and seventy nine of them were represented by *Cornus*-feeding *Phyllocnistis* and the remaining 33 specimens belonged to closely related *Phyllocnistis* species feeding on other host plants: *P. labyrinthella* (Bjerkander, 1790), *P. citrella* Stainton, 1856, *P. extrematrix* Martynova, 1955, *P. gracilistylella* Kobayashi, Jinbo & Hirowatari, 2011, *P. unipunctella* (Stephens, 1834) and *Phyllocnistis* spp. All these species are distributed in Eurasia and are known in the eastern part. The specimens of the East Asian species *P. citrella* originated from Europe and the USA (Suppl. material 1).

### *Cornus*-feeding *Phyllocnostis* were collected in the following countries:

***Japan.*** Leaf mines and individuals of *P. cornella* were sampled on Hokkaido (2 specimens) and *P. indistincta* on Honshu (137 specimens), Shikoku (1 specimen) and Kyushu (19 specimens) on different *Cornus* species (Suppl. material 1). Leaves with mining larvae and cocoon folds were sampled from March to November 2008–2017. Larvae and pupae found in mines on leaves were reared in the laboratory in plastic cups containing wet cotton, at constant conditions (20 °C, 55 % RH, LD 16:8 h photoperiod). Three larvae and six pupae were preserved in 99 % ethanol and 95 adults were pinned. In addition, specimens collected by Dr H. Kuroko in Osaka Prefecture University (OPU) and collections of N. Hirano (Matsumoto, Nagano Prefecture) were examined.

***China.*** Leaf mines of *P. saepta* with one living larva and two pupae were sampled on *C. macrophylla* in one location in Yunnan Province (Weixi) in July 2016 (Suppl. material 1). One larva and one pupa were preserved in 99 % ethanol and one adult emerged from the mine and was pinned for further studies (Suppl. material 1).

***Russia.*** Leaf mines with individuals of *P. verae* were collected on Siberian dogwood *C. alba* in Siberia (Krasnoyarsk, near village Borovoe, along the river Yenisei) in a forested area, in June–July 2015. Overall, 11 larvae were preserved in 96 % ethanol and six adults were pinned (Suppl. material 1). To obtain adults, leaves with mines were kept in glass jars at constant conditions (22 °C, 55 % RH, LD 18:6 h photoperiod), following the protocol in Ohshima (2005). In addition, 20 leaves with mines, some of them with larvae or pupae inside mines, were preserved in herbarium collections as described in Kirichenko (2014). In June–July 2015–2017, an extensive survey of *Cornus* spp. was undertaken throughout the Asian part of Russia: in Siberia – Tyumen, Sugrut, Tomsk, Omsk, Novosibirsk, Kemerovo, Barnaul, Irkutsk, Ulan-Ude and Chita, in the Russian Far East – Blagoveshensk, Vladivostok, Gornotayejnoe and on the Island Sakhalin – Yuzhno-Sakhalinsk. Dogwood trees (especially *C. alba* and *C. controversa*), growing in botanical gardens and arboreta, in city plantations and in forests, were checked for the presence of *Phyllocnistis* mines.

Photographs of leaf mines were taken in the field and in the laboratory using an OLYMPUS μ1060 digital camera (in Japan) and a digital camera Sony Nex3 (in Russia). Additionally, leaf mines of *P. indistincta* were scanned using an EPSON GT7400. Pupae of *P. indistincta* were dried under room temperature and sputter-coated with a 60:40 mixture of gold-palladium for examination with a scanning electron microscope (SEM) HITACHI SU1510 (Hitachi Ltd., Tokyo, Japan), with a lanthanum hexaboride (LaB_6_) source, at an accelerating voltage of 15 kV. Mounted moths of *Cornus*-feeding species were photographed using an OLYMPUS E-500 digital camera (*P. indistincta*). Other species were photographed using Zeiss Axiocam MRc 5 digital camera mounted on a Zeiss V.20 stereo microscope. *Phyllocnistis cornella, P. saepta* and *P. verae* specimens were photographed up to 30 times and then focus stacking was applied using Helicon Focus (http://www.heliconsoft.com/) or Zeiss Axiovision software.

Twenty five genitalia slides of *P. cornella* (two slides), *P. indistincta* (16), *P. saepta* (one) and *P. verae* (six) were prepared and analysed (Suppl. material 1). For genitalic preparation, abdomens were heated in a 10 % potassium hydroxide solution, stained with acetocarmine and then slide-mounted in Canada balsam. For *P. cornella* specimens, the abdomens were removed from the specimens and used in non-destructive DNA extraction (Doorenweerd et al. 2016). Female genitalia were stained with chlorazol black and slide mounted in euparal. Genitalia were photographed with a Leica DFC 450 digital camera through a Leitz Diaplan GMBH microscope. On the same microscope, a Leitz drawing tube was attached for drawings of genitalia. Environmental scanning electron microscope (ESEM) digital images were taken with a Hitachi TM1000. Drawing of genitalia was made by PT and SK. *P. cornella* specimens were studied with a motorised Zeiss AxioImager with Zeiss Axiocam MRc 5 camera. All images were processed in Adobe Photoshop CS5 Extended.

### Terminology

The description of forewing pattern follows Brito et al. (2017a), genitalia: Klots (1970) and Kawahara et al. (2009), pupa: Patočka and Turcani (2005). Scientific names of plants follow the Missouri Botanical Garden Tropicos database (2017) and The Plant List (2017).

### Specimen depositories

**ELKU** Entomological Laboratory, Kyushu University, Fukuoka, Japan.

**OPU** Osaka Prefecture University, Osaka, Japan.

**HAU** Hunan Agricultural University, Hunan, China.

**SIF SB RAS** Sukachev Institute of Forest, Siberian Branch of the Russian Academy of Sciences, Krasnoyarsk, Russia.

**MSNV** Museo Civico di Storia Naturale, Verona, Italy.

**RMNH** Naturalis Biodiversity Centre, Leiden, The Netherlands.

### Molecular analysis

The authors DNA barcoded 14 specimens of *Phyllocnistis* spp. feeding on *Cornus*: two specimens of *P. cornella*, five *P. indistincta*, two *P. saepta* and five *P. verae*. DNA barcodes of another 33 specimens belonging to closely related *Phyllocnistis* species (listed above, see Materials and methods) were involved to estimate interspecific distances amongst *Phyllocnistis*. Nine of them (i.e. seven specimens of *P. labyrinthella*, one *P. unipunctella* and one *Phyllocnistis* sp.1 from *Salix*) were obtained by the authors, whereas DNA barcodes of the other 24 *Phyllocnistis* specimens were taken from BOLD and/or GenBank (Suppl. material 1). All DNA barcoded *Cornus*-feeding *Phyllocnistis* specimens were additionally sequenced for two nuclear genes for comparison: histone H3 (i.e. H3) and 28S rRNA (i.e. 28S).

The following primers were used for amplification and sequencing: LCO1490 (5’ GGT CAA CAA ATC ATA AAG ATA TTG G 3’) and HCO2198 (5’ TAA ACT TCA GGG TGA CCA AAA AAT CA 3’) for the COI gene (Folmer et al. 1994), H3 F (5’ ATG GCT CGT ACC AAG CAG ACG GC) and H3 R (5’ ATA TCC TTG GGC ATG ATG GTG AC) for the H3 gene (Colgan et al. 1998) and D1F (5’ ACC CGC TGA ATT TAA GCA TAT) and D3R (5’ TAG TTC ACC ATCTTT CGG GTC) for the 28S gene (Lopez Vaamonde et al. 2001).

DNA was extracted using NucleoSpin tissue XS kit (Macherey-Nagel, Germany) according to the manufacturer’s protocol. The COI barcoding fragment (658 bp) was amplified via PCR at the standard conditions for the reaction (Hebert et al. 2003). PCR amplification with H3 gene (328 bp) was undertaken under the following conditions: 40 cycles (1 min at 94 °C, 1 min at 45 °C, 1 min at 65 °C) and with 28S (940 bp) – 30 cycles (45 s at 94 °C, 50 s at 57 °C, 1 min at 72 °C) (Kirichenko et al. 2016). PCR products were purified using the NucleoSpin Gel and PCR Clean-up kit (Macherey-Nagel, Germany) and sequenced by the Sanger method with Abi Prism Big Dye Terminator 3.1 cycle sequencing kit (25 cycles of 10 s at 96 °C, 5 s at 50 °C, 4 min at 60 °C). Sequencing was carried out using a 3500 ABI genetic analyser. The COI amplification and sequence reactions for the RMNH specimens were carried out at Naturalis Biodiversity Centre (Leiden, The Netherlands). All the remaining reactions were carried out at INRA (Orléans, France). All sequences were aligned using CodonCode Aligner 3.7.1. (CodonCode Corporation) or Geneious R6.

DNA sequences, along with the voucher data, images and trace files, were deposited in the Barcode of Life Data Systems (BOLD) (Ratnasingham and Hebert 2007) and the sequences were deposited in GenBank. All data are available in BOLD through the public dataset: dx.doi.org/10.5883/DS-PHYLCORN.

Barcode Index Numbers (BINs) (Ratnasingham and Hebert 2013) were obtained from BOLD. Intra- and interspecific genetic distances were estimated using the Kimura 2-parameter. RaxML v8 (Stamatakis 2014) was used to estimate the best-scoring tree and perform a multiparametric bootstrap test with extended majority-rule stopping criterium, following the PhylOStack v1.7 protocol (Doorenweerd 2017). As outgroups, one specimen of *Phyllonorycter connexella* (Zeller, 1846) from *Salix* sp. was used for the COI tree and one specimen of *Phyllocnistis labyrinthella* from *Populus balsamifera* L. for both the H3 and 28S analyses. Both specimens were collected in Siberia (Russia: the Republic of Khakassia, Abakan) (Suppl. material 1). Outgroups were sequenced following the protocol described above.

### Wolbachia test

Fourteen barcoded specimens of *Cornus*-feeding *Phyllocnistis* spp. were screened for infection by *Wolbachia* spp. (Rickettsiaceae). The two genes, wsp and fbpA, were amplified with the three sets of primers: (1) wspecF (5’-CATACCTAT TCGAAGGGATAG-3’), wspecR (5’-AGCTTCGAGTGAAACCAATTC-3’) and (2) wsp81F (5’-TGGTCCAATAAGTGATGAAGAAAC-3’), wsp691R (5’-AAAATTAAACGCTACTCCA-3’), for the gene wsp (respectively 438 bp and ~600bp) and (3) fbpa-F1 (5’-GCTGCTCCRCTTGGYWTGAT-3’), fbpa-R1(5’-CCRCCAGARAAAAYYACTATTC-3’), for the gene fbpA (429 bp) to test for the presence of other Rickettsiaceae following the standard protocols (Baldo et al. 2006, Kodandaramaiah et al. 2013).

PCR was run on a DNA-cycling machine 9800 Fast Thermal Cycler (Applied Biosystems – Foster City, CA). Gel electrophoresis was applied to visualise the amplified products in 1.5 % agarose gel using the gel electrophoresis apparatus (RunOne, EmbiTec, San Diego, CA). The gel was stained with ethidium bromide (EtBr) at a concentration of 0.5 μg/mL for 30 minutes (Lee et al. 2012), visualised in ultraviolet light source (wavelength 254 nm) and subsequently photographed using the image system IP-010.SD (Vilber Lourmat, France). The presence of the amplified transgene element on a gel was interpreted as evidence that the insect was infected with *Wolbachia* or related species parasite.

## Results

### DNA barcoding

**Figure 1.**
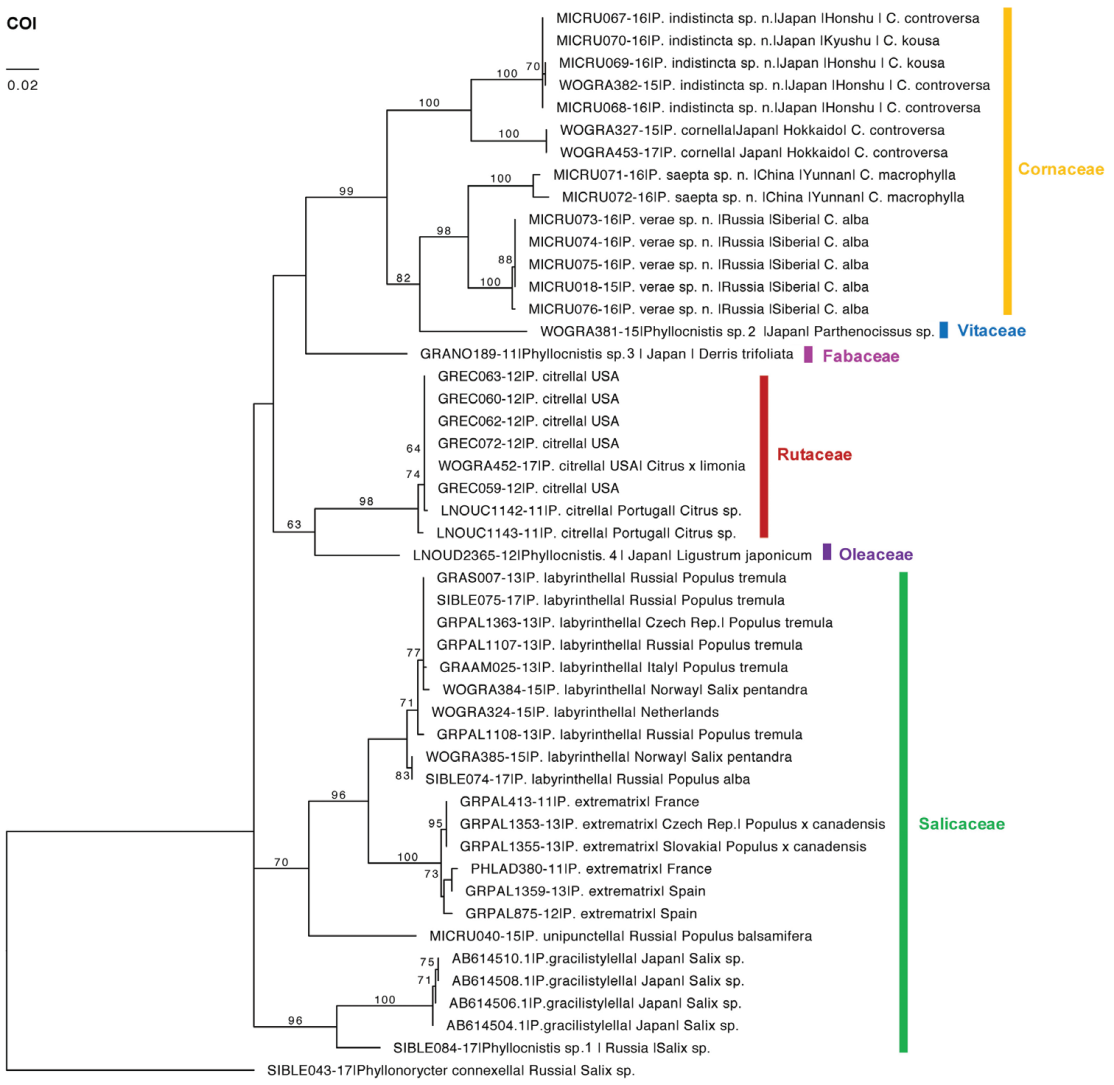
A neighbour-joining tree based on the COI barcode fragment of the 47 *Phyllocnistis* spp. specimens collected in Eurasia and beyond on woody plants from six families (indicated in the figure). Host plant genus / species is given in the figure for those leaf miners which were obtained directly from their mines; for specimens collected in other ways the host plant species remained unknown.

In total, 47 DNA barcodes of 13 *Phyllocnistis* species feeding on Cornaceae, Salicaceae, Rutaceae, Vitaceae, Fabaceae, and Oleaceae were analysed (Fig. 1). Each species had its own unique BIN in BOLD. Amongst them, there were four BINs of *Phyllocnistis* feeding on Cornaceae: one was represented by *P. cornella* (BOLD:ACU7120) and three other BINs corresponded to the new species described here: *P. indistincta* (BOLD:ACY4130), *P. saepta* (BOLD:ADE5346) and *P. verae* (BOLD:ACX7754). The other nine BINs were represented by *P. labyrinthella*, *P. extrematrix*, *P. gracilistylella*, *P. unipunctella* (all from Salicaceae), *P. citrella* (Rutaceae) and four putative new species, each from Salicaceae, Vitaceae, Fabaceae and Oleaceae (Fig. 1).

The sequences of *Cornus*-feeding species formed distinct clusters, with a maximum intraspecific divergence varying from 0 to 1.2 % versus a nearest-neighbour distance, varying from 6.1 to 13.6 %, when comparing *Cornus*-feeding species in pairs (Table 2). The minimum interspecific distance was smaller in the pairs *P. saepta* – *P. verae* (6.1 %) and *P. cornella* – *P. indistincta* (7.3 %) and doubled in comparisons across these pairs (Table 2). Diagnostic mutations separating *Cornus*-feeding species ranged from 32 to 68 (Fig. 1). No evidence for mitochondrial introgression was found.

**Table 2.** Intra- and interspecific genetic divergences in DNA barcode sequences among the studied *Phyllocnistis* species*.

**Species^1^**	***Phylocnistis cornella***	***P. indistincta***	***P. verae***	***P. saepta***	***Phyllocnistis* sp. 2**	***Phyllocnistis* sp. 3**	***P. citrella***	***Phyllocnistis* sp. 4**	***P. labyrinthella***	***P. extrematrix***	***P. unipunctella***	***Phyllocnistis* sp. 1**	***P. gracilistylella***
***Phylocnistis cornella***	[**0**]												
***P. indistincta***	7.3	[**0.2**]											
***P. verae***	12.6	11.9	[**0.3**]										
***P. saepta***	13.6	11.8	6.1	[**1.2**]									
***Phyllocnistis* sp. 2**	13.8	13.2	8.9	10.6	[−]								
***Phyllocnistis* sp. 3**	14.8	15.7	14.1	13.4	13.6	[−]							
***P. citrella***	14.9	13.9	15.5	15.4	14.9	13.0	[**0.3**]						
***Phyllocnistis* sp. 4**	15.5	15.9	14.5	16.7	14.9	12.1	8.4	[−]					
***P. labyrinthella***	16.7	13.1	14.9	14.5	13.2	10.7	10.2	12.9	[**1.4**]				
***P. extrematrix***	17.1	15.3	16.5	15.6	15.2	13.5	10.6	13.7	5.6	[**1.2**]			
***P. unipunctella***	18.9	17.8	16.3	16.4	15.7	12.0	11.8	14.3	9.9	11.8	[−]		
***Phyllocnistis* sp. 1**	19.5	18.3	18.2	19.2	16.8	12.6	11.8	11.4	13.8	14.3	12.8	[−]	
***P. gracilistylella***	20.8	18.8	19.4	17.7	17.2	14.7	11.9	12.7	14.5	15.1	15.7	6.8	[**0.3**]

*Kimura 2-parameter (K2P) distances (%) for barcode DNA sequences of the 13 analyzed species in the genus *Phyllocnistis*; minimal pairwise distances are given for each species pair; values in square brackets represent maximal intraspecific distances.^1^The four putative new species of *Phyllocnistis* sp. 1 – sp. 4 were sampled each from Salicaceae (Russia), Vitaceae (Japan), Fabaceae (Japan), and Oleaceae (Japan).[−] no data because a single specimen was sequenced.

Similar minimum interspecific distances were observed between other representatives of the genus *Phyllocnistis* developing on Salicaceae: 5.6 % in the pair *P. labyrinthella* – *P. extrematrix*, 6.8 % in *P. gracilistylella* – *Phyllocnistis* sp.1 and 9.9 % in *P. unipunctella* – *P. labyrinthella* (Fig. 1, Table 2). The maximum intraspecific distance in these species did not exceed 1.4 %.

The closest neighbour to the *Cornus*-feeding *Phyllocnistis* group turned out to be an undescribed species (i.e. *Phyllocnistis* sp. 2) collected from Vitaceae in Japan, with a minimal interspecific distance of 8.9 % between *P. verae* and sp. 2. The cluster of Salicaceae-feeding *Phyllocnistis* was another close neighbour to the cluster of Cornaceae-feeding *Phyllocnistis*, with the minimal interspecific divergence (13.1 %) observed between *P. verae* and *P. labyrinthella* (Table 2).

Besides three new species on *Cornus*, DNA barcoding data revealed four putative new species in the east: three species in Japan feeding on *Parthenocissus* (Vitaceae), *Ligustrum* (Oleaceae) and *Derris* (Fabaceae), respectively and one in the Russian Far East feeding on *Salix* (Salicaceae) (Fig. 1, Suppl. material 1).

### Nuclear genes

**Figure 2.**
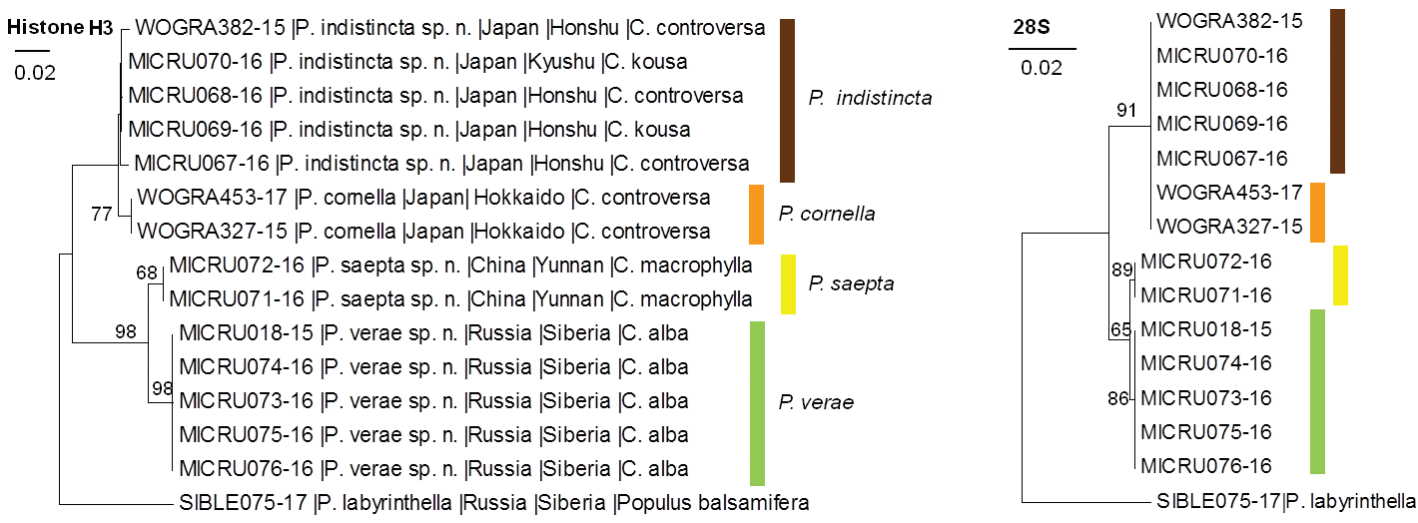
A neighbour-joining tree based on the H3 and 28S sequences in *Cornus*-feeding *Phyllocnistis* spp. from Northeast Asia.

Sequences of the nuclear genes histone H3 and 28S were obtained for previously barcoded 14 specimens of *Cornus*-feeding *Phyllocnistis* spp. (Fig. 2).

H3 explicitly delimited four clusters, corresponding to the four lineages defined by COI: *P. cornella*, *P. indistincta, P. saepta* and *P. verae*. The gene 28S supported the clusters of *P. saepta* and *P. verae*, but it did not show any divergence between *P. cornella* and *P. indistincta* (Fig. 2).

In both H3 and 28S, the number of diagnostic mutations was the least in the pairs: *P. cornella* – *P. indistincta*, (two mutations in H3 and no mutation in 28S) and *P. saepta* – *P. verae* (four mutations in H3 and two in 28S). The highest number of mutations was detected in the pair *P. cornella* – *P. saepta*, i.e. 15 and 19 mutations in 28S and H3, respectively.

### Wolbachia test

Out of the 14 specimens of the four *Cornus*-feeding *Phyllocnistis* species screened for the genes wsp and fbpA, only one specimen of *P. indistincta* (MICRU069-16) from Japan (Honshu, Nara, Tateriko, ex *Cornus kousa*) showed the presence of an infection (Suppl. material 2).

### Species descriptions

The four species described below, all feeding on *Cornus*, are morphologically very similar. The forewing pattern is characterised by a longitudinal fascia (lf), more or less defined, running along the costal margin proximally and then bent inwardly distally. Three costal and four apical ciliary strigulae are present, the first costal forming a transverse fascia (tf) crossing the wing. The male genitalia have slender valvae, rounded apically and a membranous phallus, finely pleated in the distal half, without cornuti and with a long phallobase/ductus ejaculatorius. The female genitalia have a cup-shaped antrum, a thin ductus and a large bursa with two flattened signa usually bearing a short median projection. The set of these characters allows this group of species to be differentiated quite easily from other congeners in the Palaearctic (Martynova 1955, Liu and Zeng 1989, Kobayashi et al. 2011b, Kobayashi and Hirowatari 2011) but their poor interspecific differences make identification of each species rather difficult. Recent studies (Kawahara et al. 2009, Davis and Wagner 2011) have shown that important identifying characters are sometimes present in pupae. However, only pupae of *P. indistincta* were available, so that it was not possible to use these characters. Identification keys of adults are given below, based on forewing pattern.

### Key to adults

**Table:** 

1	Forewing with inner margin of longitudinal fascia (lf) well-defined	**2**
–	Forewing with inner margin of lf indistinct, well-defined only distally; larva on *Cornus controversa*, *C. macrophylla*, *C. kousa* and *C. florida* in Honshu, Shikoku and Kyushu (Japan)	***indistincta***
2	Forewing with transverse fascia (tf) interrupted in middle; larva on *C. alba* in Siberia (Russia)	***verae***
–	Forewing with tf not interrupted	**3**
3	Forewing with strigula-shaped dark dorsal dot, distally inner margin of costal fascia connecting with short dark strigula, lf reaching tf; larva on *C. macrophylla* in Yunnan (China)	***saepta***
–	Forewing with dark dot absent or, if present, rounded; lf not reaching tf; larva on *C. controversa* in Hokkaido (Japan) and Kunashir Island (Russia)	***cornella***

#### Phyllocnistis indistincta

Taxon classificationAnimaliaLepidopteraGracillariidae

Kobayashi & Triberti sp. n.

http://zoobank.org/3B285BF9-59E0-4244-B480-8E3CEFAC0666

Figs 3 , 4 , 5 , 6 , 9A–E , 11 , 12 , 13 , 14 , 16 , 18

##### Etymology.

This Latin adjective, declined in the feminine gender, means “indistinct”. It is related to the longitudinal fascia in the forewing pattern that is basally indistinct in this species.

##### Diagnosis.

Forewing lustrous-white with a longitudinal white-yellow fascia and with an indistinct inner margin, three costal and four apical ciliary strigulae; male genitalia with phallus about as long as phallobase; female corpus bursae with two signa, similar in shape, the distal signa larger than the central signa.

Unlike other *Cornus*-feeding *Phyllocnistis*, the forewing pattern of *P. indistincta* has a well-defined outer margin of lf and an indistinct inner margin. Male genitalia differ from *P. saepta* by the phallus (about as long as phallobase or slightly shorter in *P. indistincta*); female genitalia are only distinguishable from *P. verae* by the size and the shape of the two signa.

##### Type material.

Holotype (♂): Japan: Honshu, Menashi, Imai, Soni, Uda, Nara Prefecture, 34.52N, 136.11E, 630 m, ex *Cornus controversa*, 7.VI.2008 (larva), 16.VI.2008 em., SK853, S. Kobayashi leg. (deposited in OPU).

##### Paratypes.

149 (5♂, 19♀, 125 exs). All specimens were collected in Japan.

Host *Cornus controversa*: **Honshu**, 16 exs, Nasu-Kashidoro, Nasu Imperial Villa, Yumoto, Nasu, Tochigi Prefecture, 30.IX.2006, 1–10.X.2006 em., Rear. Nos 808-1–808-10, 809-1–809-5, K. Niimi leg.; 3 exs, same locality and data, 2.VIII.2007 col., 6–8.VIII.2007 em., 982-1–982-3; 1♂ 2♀ 2exs, Okuyamada, Takayama, Nagano Prefecture, 2.VIII.2010 (larva), 2–15.VIII.2010 em., MICRU068-16, MICRU067-16, SK323♂, TRB2020♂, S. Kobayashi leg.; 1♂ 3♀, Oshirakawa, Nagawa, Matumoto, Nagano Prefecture, 17.IX.2011 (larva), 29.IX.2011 em., S. Kobayashi leg. (deposited in OPU); 4 exs, Alps Park, Matsumoto, Nagano Prefecture, 9.X.2015 (larva), 24.X.2015 em., S. Yagi & T. Hirowatari leg. (deposited in ELKU); 1 ex, Fuji-Kawaguchiko, Yamanashi Prefecture, 3.VIII.2015 (larva), 11.VIII.2015 em., S. Kobayashi leg.; 1 ex, Kirara, Dando, Shidara, Aichi Prefecture, 30.IX.2008 (larva), 6.X.2008 em., S. Kobayashi & T. Hirowatari leg.; 2♀ 4 exs, Mt. Mikusa, Nose, Osaka Prefecture, 21.V.2008 (larva), 30.V.–8.VI.2008 em., SK154, 322; S. Kobayashi & T. Hirowatari leg.; [Soni, Uda, Nara Prefecture, S. Kobayashi leg.]: 1♀, Konagao, 24.V.2008 (larva), 27.V.2008 em., SK146; 1♂ 1♀ 4 exs, Konagao, 11–8.VI.2008 (larva); 17.VI.2008 em., 1 ex, Konagao, 12.X.2008 (larva), 23.X.2008 em.; 2 exs, Kumatawa, Konagao, 30.V.2015 (larva), 3.VI.2015 em.; 1♀, Imai, 12.VII.2008 (larva), 15.VII.2008 em., SK148; 1 ex, Imai, 12.X.2008 (larva), 19–20.X.2008 em.; Menashi: 1♂ 2 exs, 23.V.2008 (larva), 3–10.VI.2008 em., SK320; 1♀ 2 exs, 7.VI.2008 (larva), 16.VI.2008 em., SK321; 1 ex, 14.VI.2008 (larva), 20.VI.2008 em.; 1 ex, 17.VI.2008 (larva), 21–24.VI.2008 em.; 1 ex, 5.VII.2008 (larva); 17.VII.2008 em.; 4 exs, 3–4.VII.2009 em., 27.VI.2009 (larva); 1 ex, 17.VI.2017 (pupa), 27.VI.2017 em. **Shikoku**, 1 ex, Fagus-no-mori, Kamagatani, Sawadani, Naka, Tokushima Prefecture, 24.VIII.2010 col., 27.VIII.2010 em., S. Kobayashi leg. **Kyushu**, Hikosan, Fukuoka Prefecture, 6 exs, 6–10.VII.1954; 7 exs, 15–26.VI.1955, H. Kuroko leg. 2 exs, Kirishima Spa., Kagoshima, 6.X.1964, H. Kuroko leg. (deposited in OPU).

Host *C. florida*: **Honshu**, [Menashi, Imai, Soni, Uda, Nara Prefecture, S. Kobayashi leg.]: 1♀, 7 exs, 26.VII.2008, 28.VII.–3.VIII.2008 em., SK164♀; 2♀ 17 exs, 27.VI.2009 (larva), 2–8.VII.2009 em., SK580 (vein); 1 ex, 19.VII.2010 (larva), 24–30.VII.2010; em.: 2 exs, Okukōchi-Sansō, Imai, 17.VI.2017 (pupa), 27.VI.2017 em., mine on upper side of bracts. **Kyushu**, Fukuoka, 1.VII.2016 (larva), 1.VII.2016 (1 adult, em. 11.VII.2016, rearing No. IsO-777) (deposited in OPU).

Host *C. kousa*: **Honshu**, 8 exs, Nasu-Kashidoro, Nasu Imperial Villa, Yumoto, Nasu, Tochigi Prefecture, 28.VII.2006 (cocoon), 5–8.VIII.2006 em., M. Murase leg.; 3 exs, 1.X.2008(larva), 7.X.2008em., Ohyoriai, Nagawa, Matsumoto, Nagano Prefecture, S. Kobayashi & T. Hirowatari leg.; Nara Prefecture: 2♀ 2 exs, Kawakami, 5.VIII.2011 (larva), 11–12.VIII.2011em., S. Kobayashi leg., slides TRB4151, TRB4153; 2exs, Tateriko, Nosegawa, 29.VII.2008 (larva), 4–18.VIII.2008 em., S. Kobayashi & T. Hirowatari leg.; Tottori Prefecture: 3exs, Mt. Daisen, 4.vii.1965, 9.VII.1965 em. (H. Kuroko), host: [Yamaboshi no Sōhō = (bracts)] (deposited in OPU).

Host *C. macrophylla*: **Honshu**, Nagano Prefecture, Matsumoto, N. Hirano leg.: 2 exs, Okada, 4–18.X.2004; 1 ex, Tomikusa, Anan, 30.VI.2006. 1♀, Tawamine, Konagao, Soni, Uda, Nara Prefecture, 24.V.2008 (larva), 27.V.2008 em. slide TRB4148, S. Kobayashi leg.; 2 exs, Same locality, 17.VII.2011 (larva), 20–25.VII.2011 em., S. Kobayashi leg.; 1 ex, Mt. Daisen, Tottori Prefecture, 4.VII.1965, 12.VII.1965, H. Kuroko leg. (deposited in OPU). **Kyushu**, 1♂, 2♀, Ito campus (Kyushu Univ.), Nishi-ku, Fukuoka, 26.V.2017 (larva), 31.V–9.VI.2017 em., S. Yagi, T. Hirowatari, K. M. M. Kyaw & C. Tsuji leg. (deposited in ELKU).

##### Additional material examined.

*Pupa* (6): **Honshu**, 1 ex, Oshirakawa, Nagawa, Matsumoto, Nagano, *Cornus controversa*, 17.IX.2011 (larva), 29.IX.2011 (preserved), S. Kobayashi leg.; Soni, Uda, Nara Prefecture, S. Kobayashi leg., *Cornus controversa*: 1 ex, Konagao, 4.VI.2011 (larva), 17.VI.2011 (preserved); 4 exs, Menashi, Imai, 17.VI.2017 (larva), 19.VI.2017 (preserved), (deposited in OPU).

*Larva* (2): **Honshu**, 1 ex, Takayama, Gifu Prefecture, *C. controversa*, 6.X.2014, RMNH.INS.30395, E.J. van Nieukerken & S. Richter leg. (deposited in RMNH); **Kyushu**, 1 ex, Mt. Hikosan, Soeda, Fukuoka, *Cornus kousa*, 1.VII.2016, rearing No. IsO-764, I. Ohshima leg. (deposited in OPU).

##### Description of adult.

(Fig. 9A–E). Wing span 5.0 mm in holotype, 4.0–6.5 mm in paratype; forewing length 2.4 mm in holotype, 2.0–3.0 mm in paratypes.

*Head.* Frons and vertex smooth, lustrous white. Antennae and labial palpi white yellowish.

*Thorax.* Tegulae, thorax and legs white. Forewing lustrous white, subapical area orange with a small apical black spot; a yellow-orange lf along costa from base to middle, margined with dark brown on both sides, then bent inwards distally, inner margin often indistinct in basal 2/3 not touching transverse fascia. Cilia white with a tf from costal 2/3 to dorsal 1/2, sometimes interrupted in the middle and three dark brown costal strigulae before apex; a black apical spot, giving origin to four divergent dark brown apical strigulae, one extending to upper part of costal cilia, the second and third to apex, the fourth to upper part of terminal cilia; terminal cilia white with a fuscous fringe line near termen. Hindwing lustrous white. This species and other members of the genus *Phyllocnistis* share an R1 vein arising from the apical half of the discoidal cell in the forewing (Fig. 13; Kawahara et al. 2017: Fig. 4C).

*Abdomen*. Mostly white yellowish dorsally, white ventrally. In the male, coremata present on segment 8, consisting of a pair of elongate, dilated extensions bearing a terminal cluster of long slender scales. In the female, dorsally on segment 8, a pair of tufts of scales longer than those covering the segment.

*Male genitalia* (Fig. 14). Tegumen elongate, touching apex of valvae, membranous, ventro-basally setose with 22 to 46 setae of varying length. Valva slender, broaded 1/4 to apex, transtilla arising from base of valva as an elongate, acute process (Fig. 14B, F); vinculum well developed, U-shaped; phallus slender, weakly sclerotised, externally finely wrinkled, about as long as phallobase or slightly shorter, cornuti absent (Fig. 14C, D).

*Female genitalia* (Fig. 16). Anterior apophyses slightly longer than posterior apophyses; ostium bursae opening in membrane between sternum 7 and 8; antrum membranous, funnel-shaped, narrowing slightly up to the size of the ductus bursae; ductus bursae completely membranous, slender, as long as antrum and terminating in the caudal area of corpus bursae; two signa are here present, usually similar in shape, the caudal bigger than central one, each with a single, short, median projection, but sometimes this projection, usually spine-shaped, is furcate or very reduced; on the wall around signa, minute scattered sclerites, thinner in the remainder of corpus bursae; ductus seminalis elongate, slightly larger than ductus bursae and arising from anterior end of corpus bursae; ductus spermathecae (not figured) with efferent canal short, forming 3 coils of equal diameter before vesicle.

##### Pupa.

(Fig. 18). Maximum length 2.8 mm, diameter 0.8 mm. Vertex with a triangular frontal process (cocoon-cutter), minutely serrated in profile and with elongated and thin apex curved towards dorsum (Fig. 18A–F); a single pair of short setae at base of frons (clypeus) (Fig. 18A); antenna extending to abdominal segment A6. A pair of relatively long setae latero-dorsally on meso- and metathorax; forewing extending to A6–7. A pair of minute dorsal setae from A1 to A8 with a second pair of large thorn-like setae, more distal, from A3 to A8, delimiting an elongated area of minute spines, projected posteriorly, from A3 to A8 (Fig. 18G–I); each abdominal segment with a pair of long, lateral, sensory setae, probably covered by the wings in A1; a pair of divergent lobate processes from caudal apex of A10 (Fig. 18J–L).

##### Biology.

**Figure 3.**
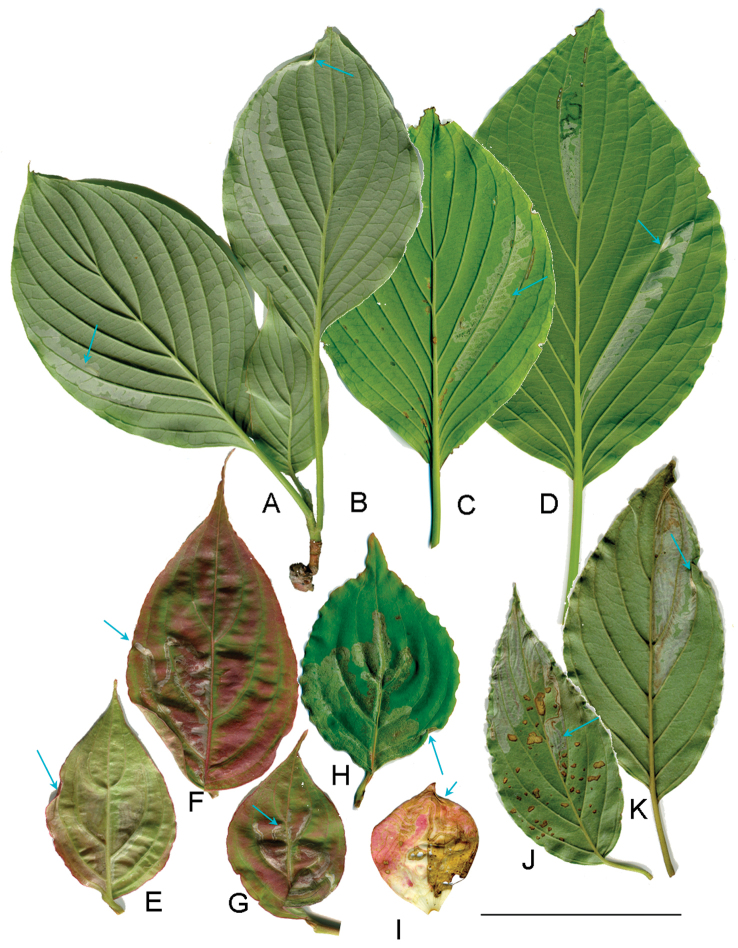
*Phyllocnistis indistincta*, mines on leaves of *Cornus* spp. in Japan (Honshu, Nara Prefecture). **A–D** *Cornus controversa* **E–I** *C. florida* **J, K** *C. macrophylla* **A, B** Konagao, Kumatawa, 670 m **C, D, H–J** type locality: Soni, Imai, Menashi, 630 m **E, F** Konagao, Tawamine, 685 m **G, K** Imai, Oku-Kochi Sanso, 455 m **I** mine on bract. Arrows show mines (**A, C, G, J**) and pupation site (**B, D, E, F, H, I, K**). Scale bar: 50 mm.

**Figure 4.**
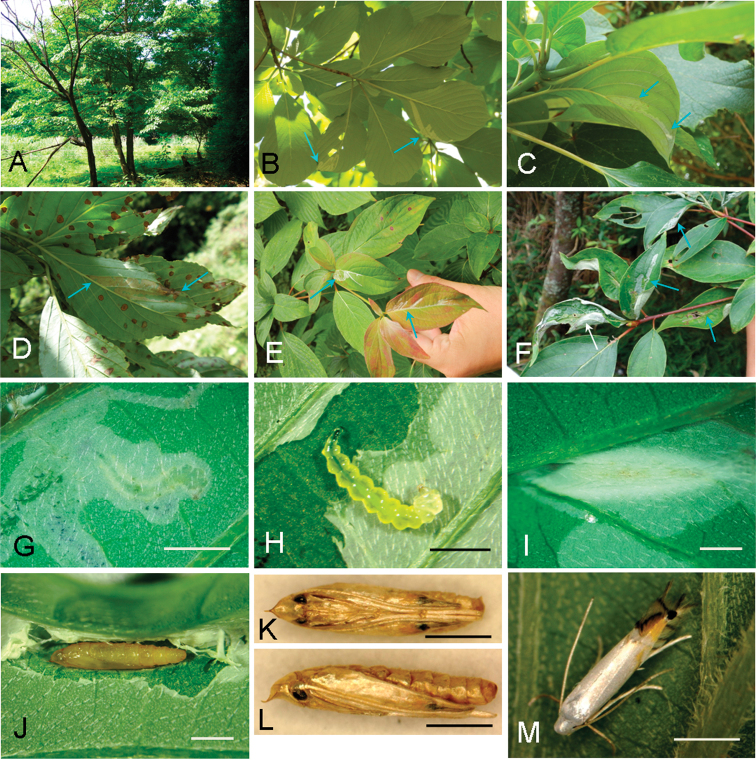
Biology of *Phyllocnistis indistincta* in Japan (Honshu) and its hostplant, *Cornus controversa*. **A, B, D, K, I** Nara Prefecture, Soni, 400–630 m **C, E** Yamanashi Prefecture, Kawaguchi-ko, 880–950 m **F–J, M** type locality: Nara Prefecture, Soni, Imai, Menashi, 630 m **A** habitat **B, C** serpentine mines, lower side of leaves **D** old mine, same side **E** young mines, upper side of leaves **F** old mines, same side **G, H** later instar larva **I** cocoon fold **J** pupa, dorsal view **K** same, ventral view **L** same, lateral sview **M** resting posture of the adult, dorsal-lateral view. Arrows show mines. Scale bars: 2 mm (**G, H**), 1 mm (**I–M**).

**Figure 5.**
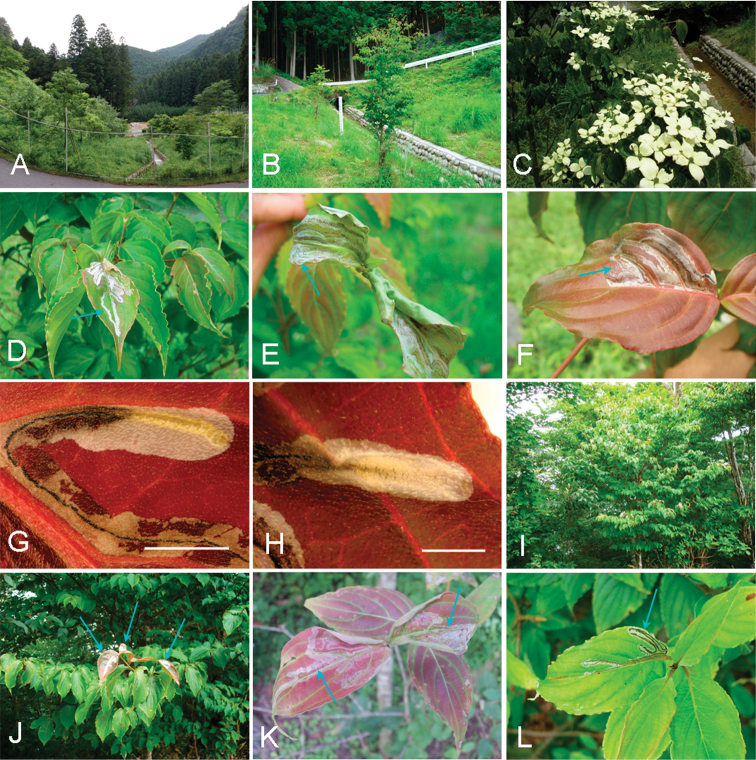
Biology of *Phyllocnistis indistincta* in Japan (Honshu) and its host plants. **A–H** type locality: Nara Prefecture, Soni, Imai, Menashi, 630 m, *Cornus florida* **I, J** Nara Prefecture,Obako-dake, 1000 m, *C. kousa* **K** Yamanashi Prefecture, Yamanaka-ko, *C. kousa*, 1000 m **L** Nara Prefecture, Soni, Konagao, 685 m, *C. macrophylla* **A** habitat **B** host plant **C** flowers **D–F, G** later instar larva **H** cocoon fold **I** host plant **J** mines and branches of host plant **K, L** serpentine mines on upper side of leaves. Arrows show mines. Scale bars: 2 mm (**G, H**).

**Figure 6.**
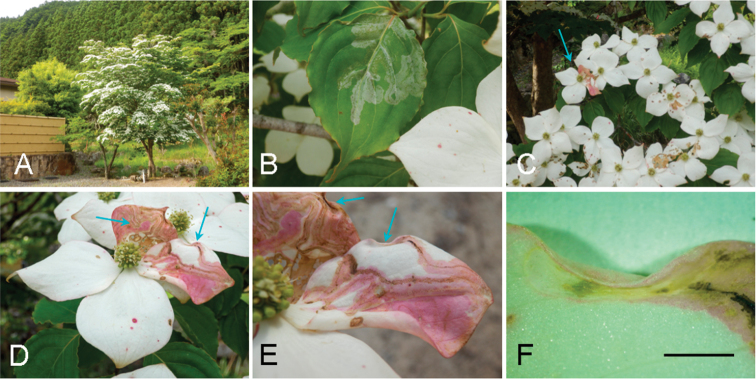
Biology of *Phyllocnistis indistincta* on *Cornus florida* in Japan (Honshu, Nara Prefecture, Soni, Imai, Oku-Kochi Sanso, 455 m). **A** habitat **B** serpentine mines on upper side of leaves **C** flowers and bracts, an arrow shows mine **D** serpentine mines on upper side of bracts **E** same, mines and cocoon folds **F** cocoon fold. Arrows show mines (**C, D**) and pupation site (**D, E**). Scale bar: 2 mm (**F**).

(Figs 3–6). The larvae mine leaves of *Cornus* species, forming a long serpentine mine parallel to leaf veins (Figs 3, 5D–F and L); about 10 cm in length, 2.0–5.0 mm in width, with a brownish frass line 1.0 mm in width. There were one to two mines per leaf (Figs 3, 5D and J). The mines were usually observed on the lower side of leaf on *C. controversa* (Figs 3A–D, 4B–D), more often on upper side of leaf on *C. florida* and *C. kousa* (Fig. 5D–F, J–L). Late instar larvae are about 4.0–6.0 mm long and pale yellow in colouration (Fig. 4H). The final instar spins a white cocoon at the leaf margin, the leaf margin slightly curled upwards by contraction of the cocoon silk. The pupal cocoon fold is 5.0 mm in length, 1.0–1.1 mm in width (Fig. 4I). Dr H. Kuroko collected adult moths of this species in Mt. Daisen, Tottori Prefecture, from flower bracts with mines containing larvae. In the present study, two bracts were observed of *C. florida* with similar serpentine mines (pale pink to ocherous, about 15 cm in length, 0.5–2.0 mm in width, brownish frass line: 0.1–0.2 mm in width) (Figs 3K, 6C–E). A pupal cocoon fold (whitish to ocherous, 5.7 mm in length, 1.0 mm in width) was formed along the bract margin (Fig. 6F). A mine on the upper side of leaf was observed in the same tree (Fig. 6B).

In the Nasu Imperial Villa of Tochigi Prefecture, Arita et al. (2009) collected this species as *Phyllocnistis* sp. 1, “Mizuki-Kohamoguri” and noted some aspects of forewing pattern and leaf mine on *Cornus controversa* and *C. kousa*.

##### Phenology.

In Japan in 2008–2016, larvae were observed from June to October. The overwintering form of this species is unknown.

##### Ecology and host plants.

(Fig. 5A, B). The host plants are *Cornus controversa*, *C. florida, C. macrophylla* and *C. kousa*.

##### Distribution.

Japan: Honshu (Tochigi, Nagano, Yamanashi, Aichi, Nara, Osaka and Tottori Prefectures), Shikoku (Tokushima Prefecture), Kyushu (Fukuoka and Kagoshima Prefectures).

##### Remarks.

In the type series of *P. indistincta*, some specimens have a forewing pattern which differs in the following points: 1) a well-defined inner margin of lf (Figs 11C, F, 12C, E–G, K), 2) a fuscous to dark orange dorsal spot at dorsum 1/4 (Figs 11C, F, 12F), 3) tf is indistinct or interrupted in the middle (Figs 11C, 12B, C, G and I), i.e. more similar to that of the other *Cornus*-feeding *Phyllocnistis*. The forewing pattern differences were recorded in the type locality of this species, Menashi, Nara Prefecture (Figs 11D, E, 12A–C), in Ohshirakawa, Nagano Prefecture (Figs 11C, F, 12E) and in Nasu Imperial Villa, Tochigi Prefecture (Fig. 12F; see also Arita et al. 2009: Pl. 3, Fig. 17). Judging from the genital characters, the differences in forewing pattern are treated as individual variation. The Indian and Japanese species, *P. toparcha* has a similar forewing pattern, with individual variation as *P. indistincta*, but *P. toparcha* is separated from *P. indistincta* by well-defined inner margin of strigulae and rather large lf. Accoding to Kuroko (1982), the autumn generation of *P. toparcha* has bolder strigulae and scattering fuscous to dark scales from base to dorsum 1/2 than the summer generation, but SK collected adult moths of *P. toparcha* having a dark dorsal spot or blotch and a darker patch in the lower half of the apical portion in summer (Kobayashi, unpublished data).

#### Phyllocnistis verae

Taxon classificationAnimaliaLepidopteraGracillariidae

Kirichenko, Triberti & Lopez-Vaamonde sp. n.

http://zoobank.org/6B89134B-B291-4C82-B7C4-AC62296C043E

**Figure 7.**
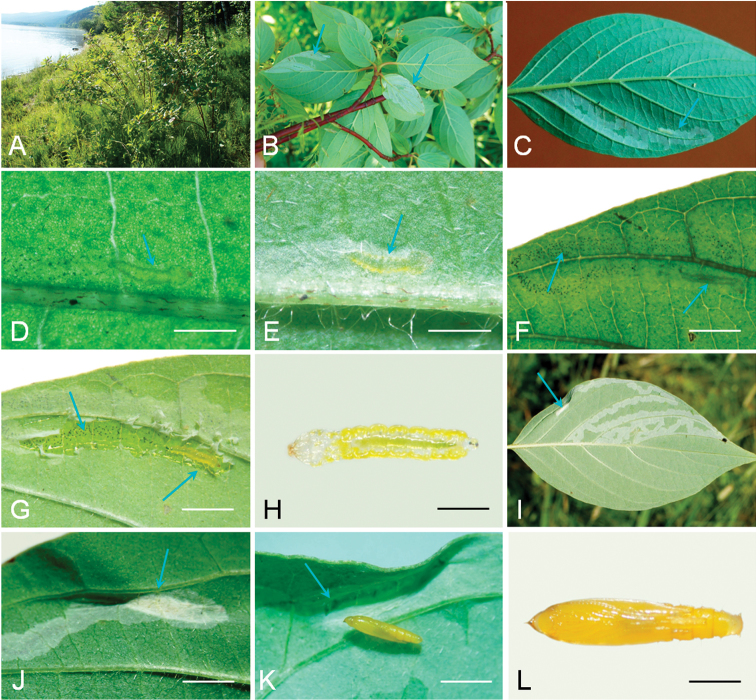
Biology of *Phyllocnistis verae* on *Cornus alba* in Russia (type locality: Krasnoyarsk, village Borovoe, left bank of Yenisei River, 144 m). **A** habitat **B** branch with mined leaves on the lower side **C** mine with feeding larva **D** fragment of mine with young larva (transmitted light) **E** same, incident light **F** line of frass and feeding larva (transmitted light) **G** opened mine **H** sap-feeding larvae, dorsal view **I, J** pupation near leaf margin **K, L** pupa. Arrows show mines (**B, C**), larva (**D, E, F, G**), frass (**G, F**), pupation site (**I–K**). Scale bars: 3 mm (**D, E**), 5 mm (**F, G**), 1 mm (**H, L**), 2 mm (**J, K**).

Figs 7 , 10A, B , 15A–C , 17A–C

##### Etymology.

The species name, *verae* is a patronym in commemoration of Mrs. Vera Kirichenko, the mother of the first author.

##### Diagnosis.

Forewing lustrous-white with a complete lf, three costal and four apical ciliary strigulae, tf interrupted; male genitalia with phallus shorter than phallobase; female corpus bursae with two signa, similar in size and shape.

Forewing pattern of *P. verae* is distinguished from *P. saepta* and *P. cornella* by the interrupted tf. In male genitalia, length of the phallus and the phallobase is similar to *P. indistincta*, but with a higher number of ventral setae (42–50). In the female genitalia, the two signa are very similar in shape and size, while they are different in the other *Cornus*-feeding species.

##### Type material.

Holotype (♂): Russia, Krasnoyarsk, near village Borovoe, along the river Yenisei (left bank), rock (skala) Berkut, 55.97N, 92.55E, 144 m, ex *Cornus alba*, 7.VII.2016 (larva), 14.VII.2016 em., No. 6-3, TRB4200, N. Kirichenko leg. (deposited in SIF SB RAS).

##### Paratypes.

(5): 4♂, TRB4116, TRB4152, TRB4199, TRB4225; 1♀, Russia, Krasnoyarsk, near village Borovoe, along the river Yenisei, rock (skala) Berkut, ex *Cornus alba*, 28.VI.2015, TRB4116, N. Kirichenko leg. (deposited in MSNV).

##### Additional material examined.

Larvae (11): Russia, Krasnoyarsk, near village Borovoe, along the river Yenisei, rock (skala) Berkut, *Cornus alba*, 3 larvae, 28.VI.2015, 4 larvae, 5.VII.2016, 4 larvae, 7.VII.2016, N. Kirichenko leg. (deposited in SIF SB RAS).

##### Description of adult.

(Figs 10A and B). Wing span 6.0–6.1 mm (6.1 mm in holotype).

*Head, thorax, legs and hindwing* do not differ from the other *Cornus*-feeding species. Forewing lustrous white, subapical area orange with a small dark spot; lf well-defined on both sides; cilia white with tf always interrupted in the middle, three dark brown costal and four apical strigulae.

*Abdomen*. Like in *cornella*.

*Male genitalia* (Fig. 15A–C). Tegumen elongate, slightly passing the apex of valvae, ventro-basally with 42-50 setae of variable length; valva not differing from the other species; phallus slender, membranous, finely wrinkled, shorter than phallobase, cornuti absent.

*Female genitalia* (Fig. 17A–C). The whole structure is similar to the other species. Bursa copulatrix with two flattened signa bearing usually a short median projection, both very similar in shape and size.

##### Pupa.

Not studied.

##### Biology.

(Fig. 7). The mine is similar to that of other *Phyllocnistis* species: a very long serpentine subepidermal tunnel, slightly widening to the end, not intersecting itself (Fig. 7B, C, I). Black grains of frass form a rather wide central line (Fig. 7F). The mine is on the lower side of the leaf, often following secondary veins, crossing them closer to the leaf margin where veins are thinner (Fig. 7B–G). Young and late instar larvae are yellow (Fig. 7D–H). The tunnel ends upon the leaf margin or 10–15 mm away from it, where the mine slightly widens (Fig. 7I, J). Here, silk is deposited across the tunnel that causes contraction of this part of the mine, where pupation takes place (Fig. 7K–L).

##### Phenology.

In 2015, by the 5th of July, when insect mines were found in nature, most larvae were at their final stage and some already had pupated. It suggests that larval development of the first generation may have started in late May. Thus, adults of the first generation can be on the wing in mid July. There are no records of the second generation. The overwintering stage remains unknown.

##### Ecology and host plants.

(Fig. 7A). In Central Siberia, the moth inhabits the forested areas. In Krasnoyarsk, mines were found on bushes growing along the river (Fig. 7A). The host plant is *Cornus alba*, the only native *Cornus* species in Siberia (Koropachinskiy and Vstovskaya 2012).

##### Distribution.

Russia, Siberia. Occurs in the southern part of Krasnoyarsk Kray, in the suburb of Krasnoyarsk. In 2015–2017, no mines were found on *Cornus* spp. in other regions of Asian Russia (Tyumen, Omsk, Novosibirsk Oblasts, Khanty-Mansi Autonomous Okrug, Tomsk, Kemerovo, Irkutsk Oblasts, Altai Kray, the Republic of Buryatia and Transbaikalia), nor in the Russian Far East (Amur Oblast, Primorskiy Kray, the Island Sakhalin).

#### Phyllocnistis saepta

Taxon classificationAnimaliaLepidopteraGracillariidae

Kirichenko, Ohshima & Huang sp. n.

http://zoobank.org/944A8697-55AE-4D4F-8A5D-2D640FCF44A3

**Figure 8.**
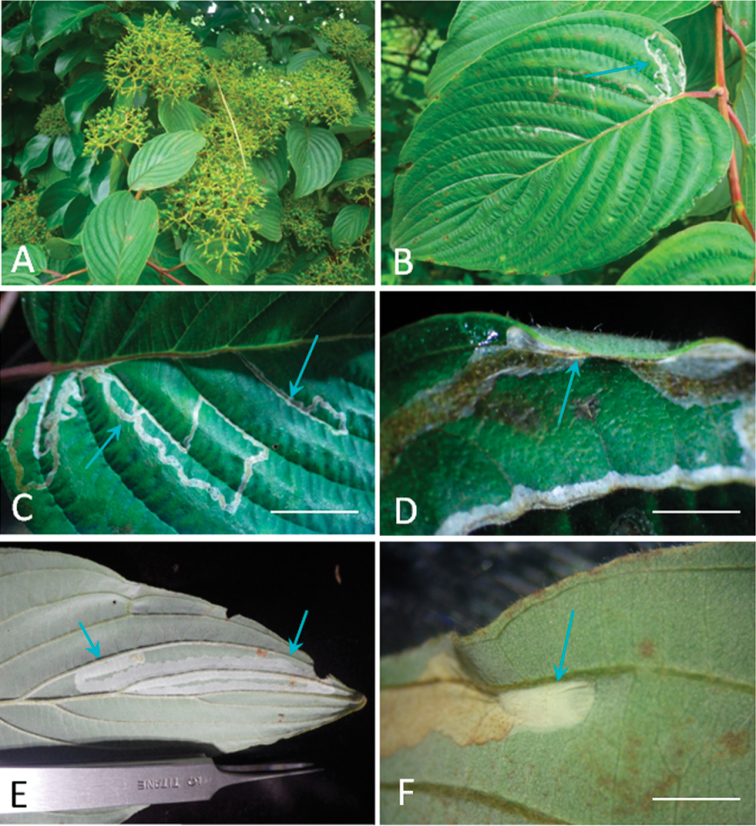
Biology of *Phyllocnistis saepta* on *Cornus macrophylla* in China (type locality: Yunnan Province, Weixi, 2800 m). **A** host plant **B, C** serpentine mine on the upper side of the leaf **D** pupation site under leaf fold on the upper side of the leaf **E** serpentine mine on the low side of the leaf **F** pupation site between two veins at a leaf margin. Arrows show mine (**B, C, E**), pupation site (**D, F**). Scale bars: 18 mm (**C**), 4 mm (**D**), 8 mm (**F**).

**Figure 9.**
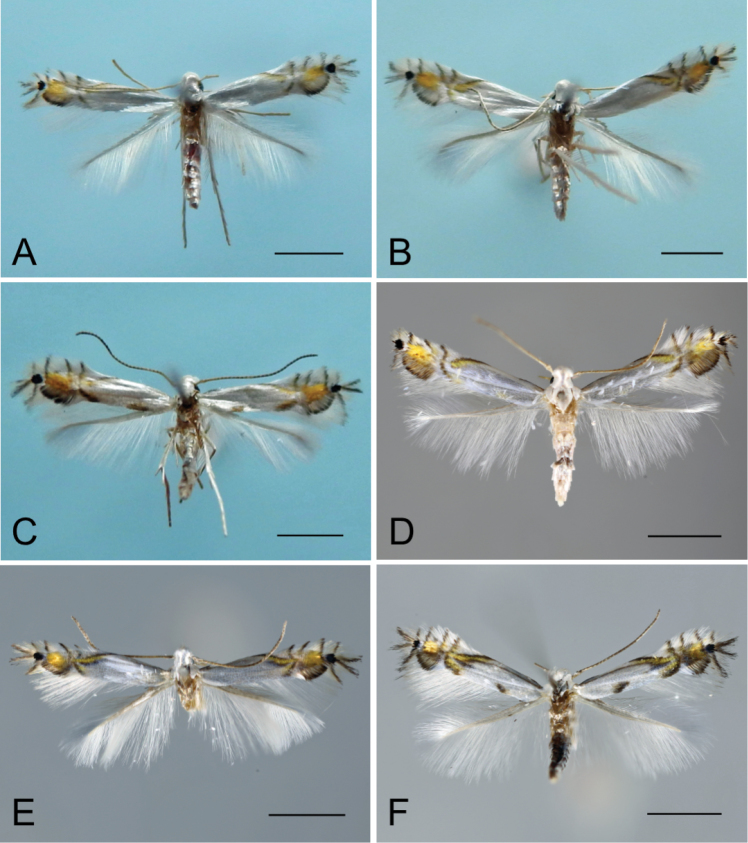
Adults of *Phyllocnistis indistincta* (Japan: Honshu, Kyushu) and *P. saepta* (China). **A**–**E** *P. indistincta* **A** holotype male, ex *Cornus controversa*, Honshu, Nara Prefecture [Suppl. material 1, No. 3] **B** paratype female, ex *C. florida*, Nara Prefecture [Suppl. material 1, No. 92] **C** paratype male, ex *C. controversa*, Nagano Prefecture [Suppl. material 1, No. 38] **D** ex *C. kousa*, Kyushu, Fukuoka Prefecture [Suppl. material 1, No. 133] **E** paratype female, Nara Prefecture [Suppl. material 1, No. 127] **F** *P. saepta*, holotype male, ex *C. macrophylla*, Yunnan Province [Suppl. material 1, No. 160].

Figs 8 , 9F , 15D, E

##### Etymology.

The name *saepta* is the past participle of the Latin verb *saepio*, that means “to block” and refers to the strigulae-shaped blotch present on the dorsum of the forewing.

##### Diagnosis.

Forewing lustrous white, lf well-defined, touching tf without interrupting it, a dorsal dark brown blotch, strigula-shaped, is present in the first third, while the inner margin of lf, apically, shows a hint of a dark line along the cell; male genitalia with a small number of ventral setae (14) and phallobase about twice the length of phallus.

*P. saepta* is distinguished from other *Cornus*-feeding species by the presence of a strigula-shaped blotch on the dorsum of the forewing. It also differs from *P. cornella* by a long lf, touching tf, from *P. verae* by tf not interrupted and from *P. indistincta* by well-defined lf.

##### Type material.

Holotype (♂): China, Yunnan, Weixi County, Diqing city, 27.16N, 99.26E, 2800 m, ex *Cornus macrophylla*, 19.VII.2016 (larva), 22.VII.2016 em., TRB4256, No. IsO-793, G.H. Huang & M. Wang leg. (deposited in HAU).

##### Additional material examined.

Larva (1) and pupa (1): China, Yunnan, Weixi County, Diqing city, *Cornus macrophylla*, 18.VII.2016 col., Nos IsO-790 and IsO-790-bis (deposited in HAU).

##### Description of adult.

(Fig. 9F). Wing span 5.0 mm.

*Head.* Like *P. indistincta*.

*Thorax.* Tegulae and thorax white, legs not present. Forewing lustrous white, subapical area orange with a small apical black spot; lf well-defined, touching tf without interrupting it. A dorsal dark brown blotch, strigula-shaped, is present in the first third, while the inner margin of lf, apically, shows a hint of a dark line along the cell (Fig. 9G). Cilia white, tf not interrupted in the middle, three dark brown costal and four apical strigulae. Hindwing lustrous white.

*Abdomen.* Not studied.

*Male genitalia* (Fig. 15D, E). Tegumen elongate, membranous, ventro-basally setose with 14 setae of varying length. Valva slender, broaded 1/4 to apex, transtilla arising from base of valva as an elongate, acute process; vinculum U-shaped; phallus slender, weakly sclerotised, externally finely wrinkled, about half the phallobase, cornuti absent.

*Female genitalia.* Unknown.

##### Pupa.

Not studied.

##### Biology.

(Fig. 8). The mine is similar to that of other *Phyllocnistis* species, on lower or upper side of the host leaf, often following secondary veins, crossing them closer to the leaf margin (Fig. 8B, C, E). Pupation takes place in the mine near leaf margin (Fig. 8D, F).

##### Phenology.

In 2016, in China (Yunnan) late instar larvae were found at the end of July.

##### Ecology and host plants.

(Fig. 8A). In China, the moth inhabits a forested area. Host plant is *C. macrophylla*.

##### Distribution.

Only one location is known so far in China – Yunnan Province, Weixi.

#### Phyllocnistis cornella

Taxon classificationAnimaliaLepidopteraGracillariidae

Ermolaev, 1987

**Figure 10.**
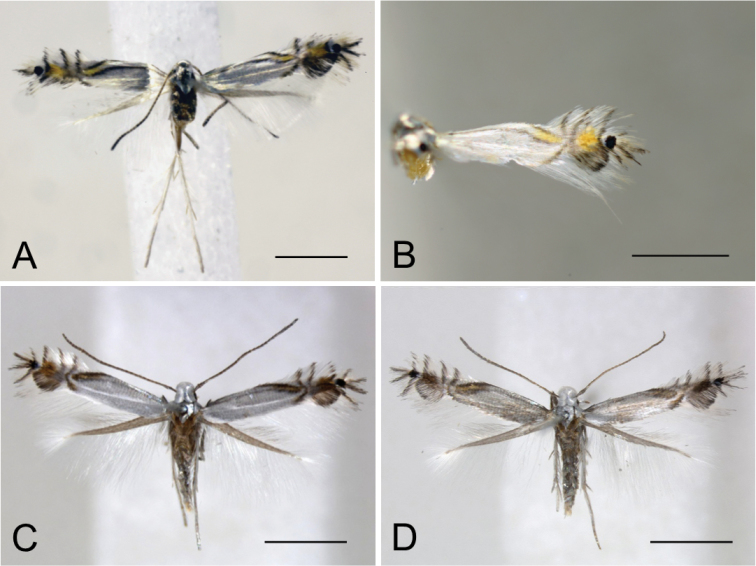
Adults of *Phyllocnistis verae* (Russia) and *P. cornella* (Japan: Hokkaido). **A**–**B** *P. verae*, ex *Cornus alba*, Krasnoyarsk **A** holotype male [Suppl. material 1, No. 163] **B** paratype female [Suppl. material 1, No. 164] **C**–**D** *P. cornella*, ex *C. controversa*, Sorachi, Kamifurano **C** female [Suppl. material 1, No. 1] **D** female [Suppl. material 1, No. 2]. Scale bar: 1 mm.

Figs 10C–D , 15F , 17D–E

Phyllocnistis cornella Ermolaev, 1987: 39–40; Seksyaeva, 1997: 429.

##### Diagnosis.

Forewing lustrous white, lf from base to 1/2, then bent inwards, not touching tf, inner margin indistinct basally; in female genitalia, bursa copulatrix with two signa usually similar in shape or smaller, with a more strongly curved median projection, the caudal signum up to twice as large as the central one.

*P. cornella* is very similar to *P. saepta*. It mainly differs in the forewing pattern, with lf not reaching tf. Hokkaido specimens show a similar forewing pattern. Unlike *P. verae*, tf is not interrupted. The male genitalia, drawn by Ermolaev, do not show any particular differential characters. Females are indistinguishable from *P. indistincta*, but differ from *P. verae* by size and shape of signa.

##### Type material.

The type series of *P. cornella* Ermolaev comprised 21 specimens (Ermolaev 1987). Holotype (♂): Russia, Kunashir Island, Alekhino, ex *Cornus controversa* Hemsl., 25.VIII.1984, V.P. Ermolaev col. The holotype is unreachable for investigation (see Remarks).

##### Paratypes.

(20): 2♂, 12♀, Russia, Kunashir Island, Alekhino, ex *C. controversa*, 24–25.VIII.1984; 3♂, 3♀, Russia, Kunashir Island, Tretyakovo, ex *C. controversa*, 25–27.VIII.1984, V.P. Ermolaev leg. The paratypes are unreachable for investigation (see Remarks).

##### Material examined.

(Figs 10D, D, 17 D, E) 2♀, Japan Hokkaido Kamifurano, roadside of rural area with old crop patches, 43.68N, 142.36E, 211 m, ex *C. controversa*, 22.IX.2013 (pupa), 24.IX.2013 em., [Dried host voucher in RMNH collection under CD13049], RMNH.5013759, genitalia slide CDGP0275; RMNH.5007963, genitalia slide CDGP0136, C. Doorenweerd & C. van den Berg leg. (deposited in RMNH).

**Figure 11.**
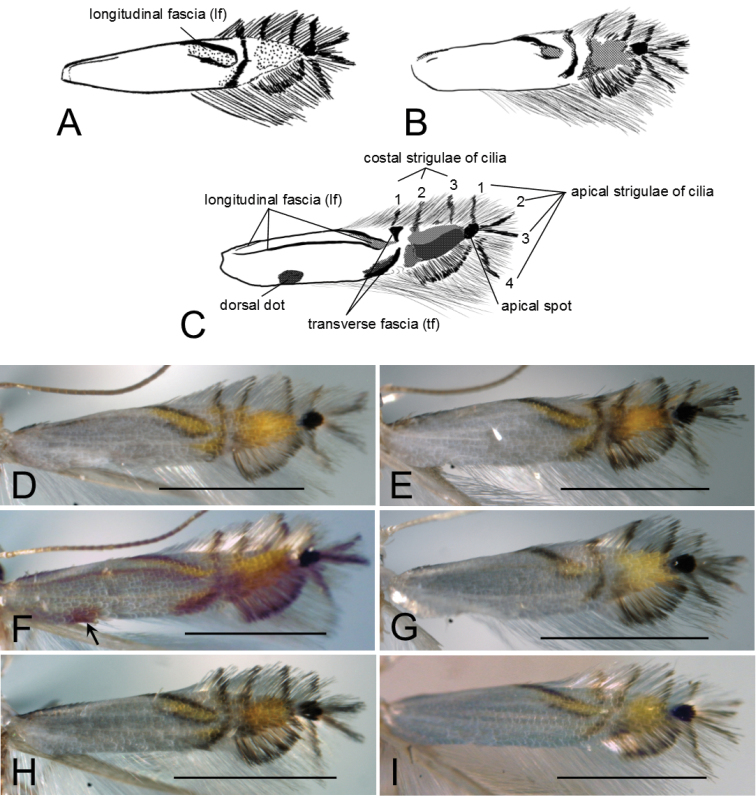
Forewing colouration and patterns of *Phyllocnistis indistincta* from *Cornus florida, C. controversa* and *C. kousa* (Japan: Honshu). **A, D, E** ex *Cornus florida* **B, C, F, G** ex *C. controversa* **H** ex *C. kousa* **I** adult emerged from mined bract of *C. florida* **A, D, E** type locality: Nara Prefecture, Soni [Suppl. material 1, Nos 93 (**A, D**) 111 (**E**)] **B, G** Osaka Prefecture, Mt. Mikusa [Suppl. material 1, No. 46] **C, F** Nagano Prefecture, Ohshirakawa [Suppl. material 1, No. 38] **H** Nara Prefecture, Kawakami [Suppl. material 1, No. 126] **I** Tottori Prefecture, Mt. Daisen [Suppl. material 1, No. 129]. Arrows show variable characters. Scale bars: 1 mm.

**Figure 12.**
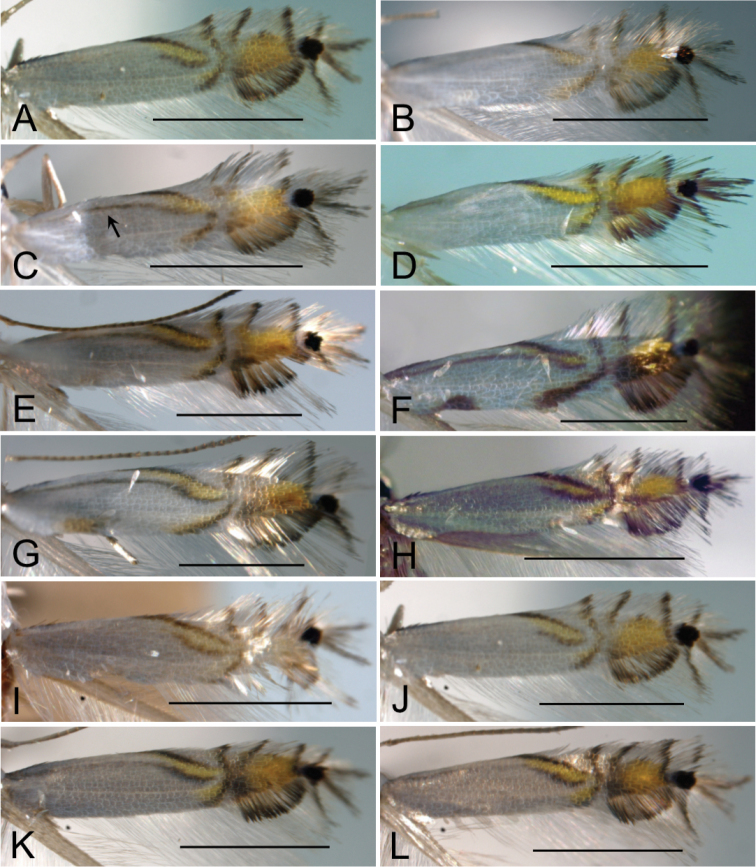
Forewing colouration and patterns of *Phyllocnistis indistincta* from *Cornus controversa* and *C. macrophylla* (Japan: Honshu, Shikoku, Kyushu). **A–I** ex *C. controversa* **J–L** ex *C. macrophylla* **A–C** type locality, Honshu, Nara Prefecture, Soni, Imai, Menashi [Suppl. material 1, Nos 67 (**A**), 69 (**B**), 71 (**C**)] **D** Yamanashi Prefecture, Fuji-Kawaguchiko [Suppl. material 1, No. 44] **E** Nagano Prefecture, Ohshirakawa [Suppl. material 1, No. 37] **F** Tochigi Prefecture, Nasu Imperial Villa [Suppl. material 1, No. 14] **G** Aichi Prefecture, Kirara [Suppl. material 1, No. 45] **H** Shikoku, Tokushima Prefecture, Naka [Suppl. material 1, No. 141] **I** Kyushu, Fukuoka Prefecture, Hikosan [Suppl. material 1, No. 142] **J**–**L** Honshu, Nagano Prefecture, Okada [Suppl. material 1, Nos 135 (**J**), 133 (**K**), 134 (**L**)]; Arrows show variable characters. Scale bars: 1 mm.

**Figure 13.**
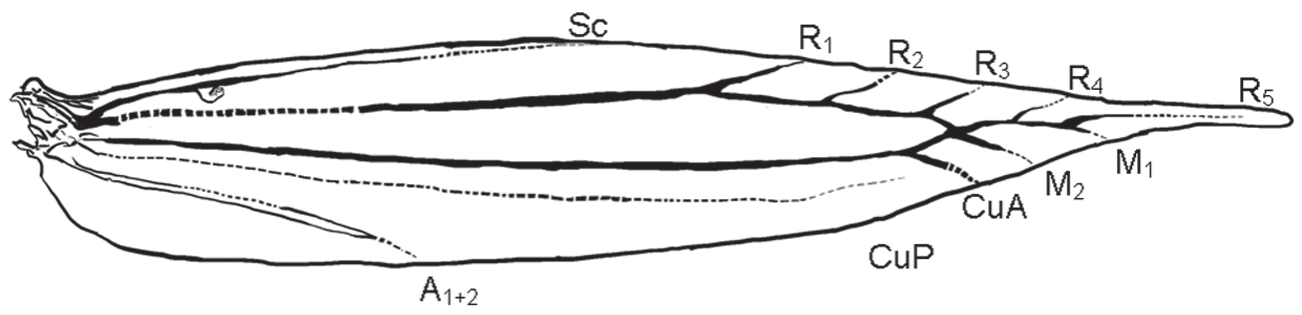
Forewing venation of *Phyllocnistis indistincta*, ex *Cornus florida*, Japan (type locality: Honshu, Nara Prefecture, Soni, Imai, Menashi), wing vein slide SK580 [Suppl. material 1, No. 108].

##### Description of adult.

(Fig. 10C, D). Wing span 4.6–5.1 mm.

*Head.* Like *P. indistincta*.

*Thorax.* Tegulae, thorax and legs white. Forewing lustrous white, subapical area copper-coloured with a small apical black spot; lf from base to 1/2, then bent inwards, not touching tf, inner margin indistinct basally. Cilia white, tf not interrupted, three dark brown costal and four apical strigulae (Fig. 10C, D). Hindwing lustrous white.

*Abdomen.* As in *P. indistincta*.

*Male genitalia* (Fig. 15F). From the Ermolaev’s figure, it is not possible to see any differential character. The authors did not have any male specimen from Hokkaido.

**Figure 14.**
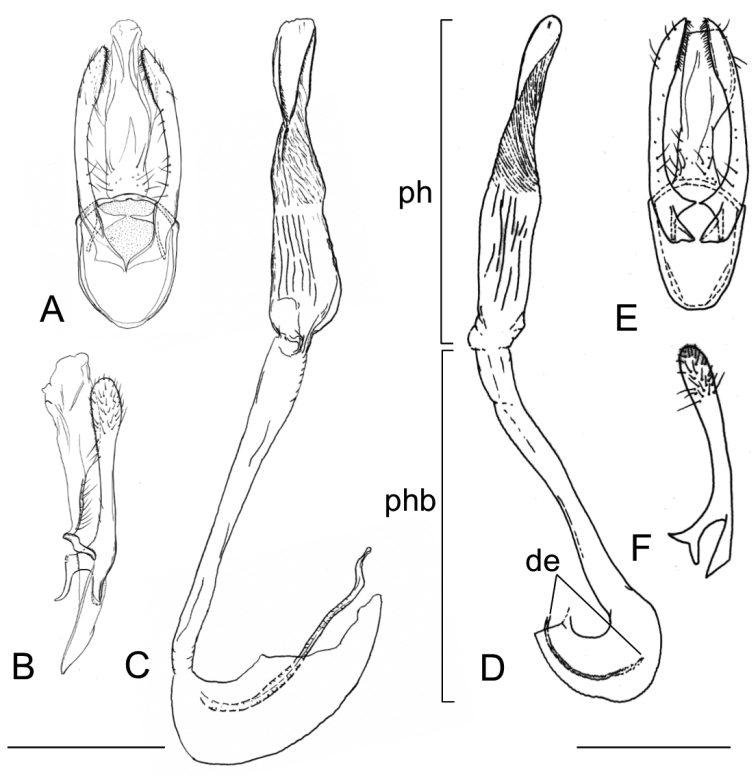
Male genitalia of *Phyllocnistis indistincta* (Japan: Honshu). **A–C** paratype, ex *Cornus controversa*, Nara Prefecture, gen. slide SK163 [Suppl. material 1, No. 52] **D–F** paratype, ex *C. controversa*, Nagano Prefecture, gen. slide TRB4220 [Suppl. material 1, No. 33] **A, E** ventral view **B, F** lateral view **C, D** phallus **B, F** left valva. Scale bars: 0.2 mm (under **B** also refers to **A, C**, under **D** also refers to **E**, **F**).

*Female genitalia* (Fig. 17D, E). Very similar to *P. indistincta*. Bursa copulatrix with two signa usually similar in shape, or the smaller with a more strongly curved median projection, the caudal signum up to twice as long as the central one (Fig. 14D, E); both signa have a spine-shaped median projection, about as long as the signum. On the wall around signa, minute scattered sclerites, thinner on the remainder of corpus bursae; ductus seminalis elongate, slightly longer than ductus bursae and arising from anterior end of corpus bursae.

**Figure 15.**
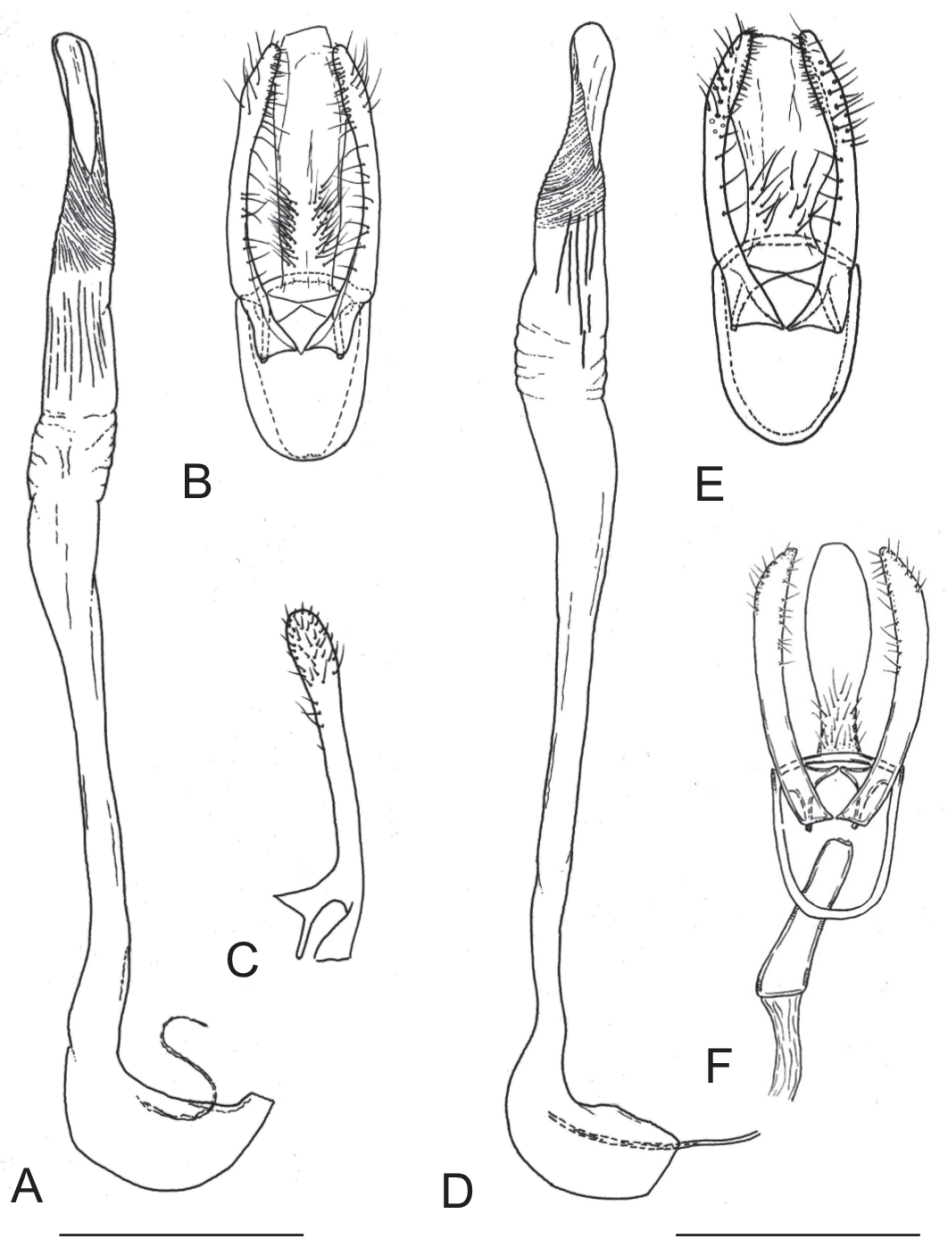
Male genitalia of *Phyllocnistis verae* (Russia), *P. septae* (China), and *P. cornella* (Russia). **A–C** *P. verae*, holotype, ex *Cornus alba*, Krasnoyarsk, gen. slide TRB4200 [Suppl. material 1, No. 163] **D, E** *P. saepta*, holotype, ex *C. macrophylla*, Yunnan, gen. slide IsO-793 [Suppl. material 1, No. 160] **F** *P. cornella*, holotype, ex. *C. controvera*, Kunashir, drawing from Ermolaev (1987) **B, E, F** ventral view **C** lateral view **A, D** phallus **C** left valva. Scale bars: 0.2 mm (under **A** also refers to **B, C** under **D** also refers to **E, F**).

**Figure 16.**
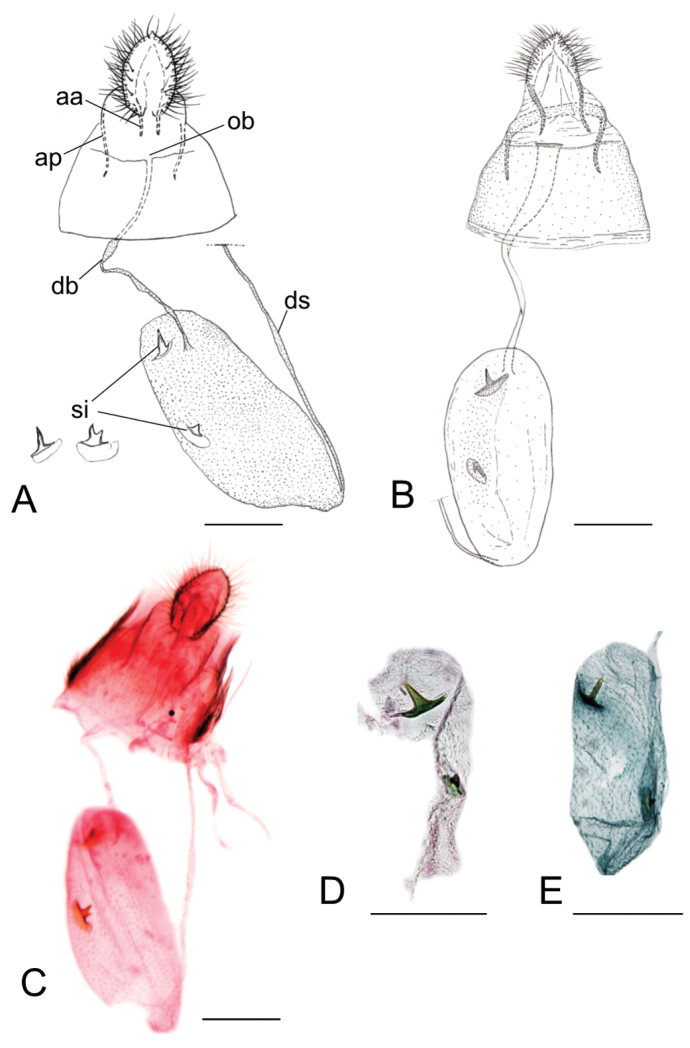
Female genitalia of *Phyllocnistis indistincta* (Japan: Honshu, Nara Prefecture). **A, C** paratype, ex *Cornus controversa*, gen. slide SK146 [Suppl. material 1, No. 51] **B** *C. macrophylla*, gen. slide TRB4148 [Suppl. material 1, No. 137] **D** ex *C. kousa*, gen. slide TRB4151 [Suppl. material 1, No. 128] **E** ex *C. kousa*, gen. slide TRB4153 [Suppl. material 1, No. 127]. Abbreviations: **ap** apophysis posterioris **aa** apophysis anterioris **db** ductus bursae **ds** ductus seminalis **ob** ostium bursae **si** signum. Scale bars: 0.2 mm.

**Figure 17.**
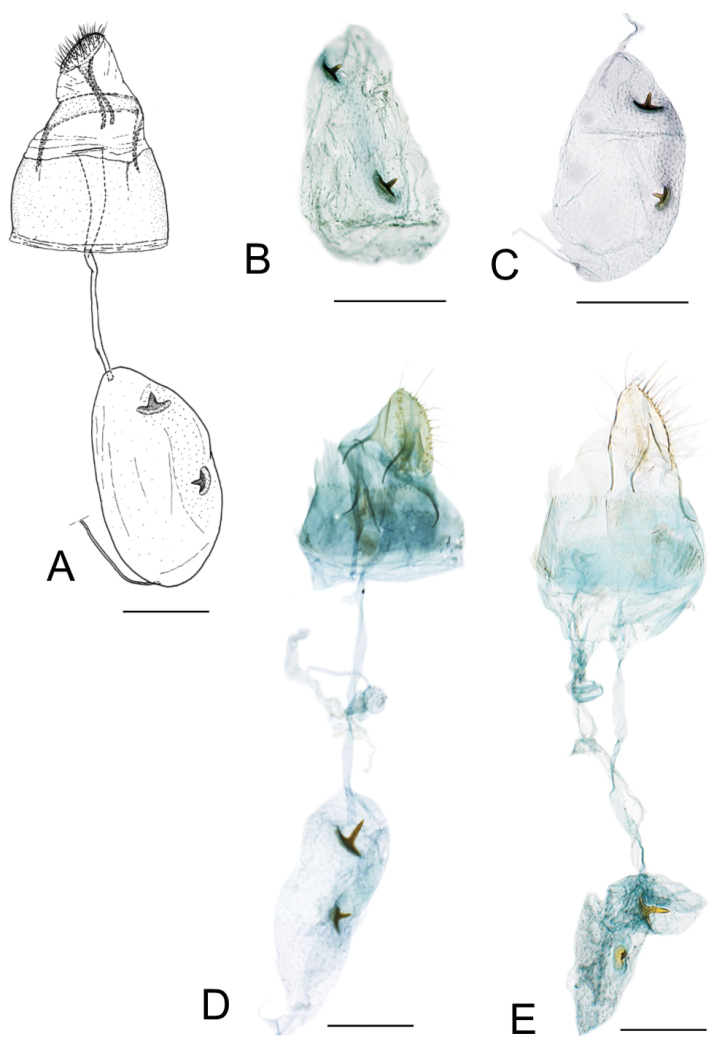
Female genitalia of *Phyllocnistis verae* (Russia) and *P. cornella* (Japan: Hokkaido). **A–C** *P. verae* **A** paratype, ex *Cornus alba*, Krasnoyarsk, gen. slide TRB4116 [Suppl. material 1, No. 164] **B** paratype, ex *C. alba*, Krasnoyarsk, gen. slide TRB4152 [Suppl. material 1, No. 166] **C** paratype, ex *C. alba*, Krasnoyarsk, gen. slide NK-39-15-4 [Suppl. material 1, No. 168] **D, E** *P. cornella*, ex *C. controversa*, Sorachi, Kamifurano, gen. slides CDGP0275 and CDGP0136 [Suppl. material 1, Nos 1–2]. Scale bars: 0.2 mm.

**Figure 18.**
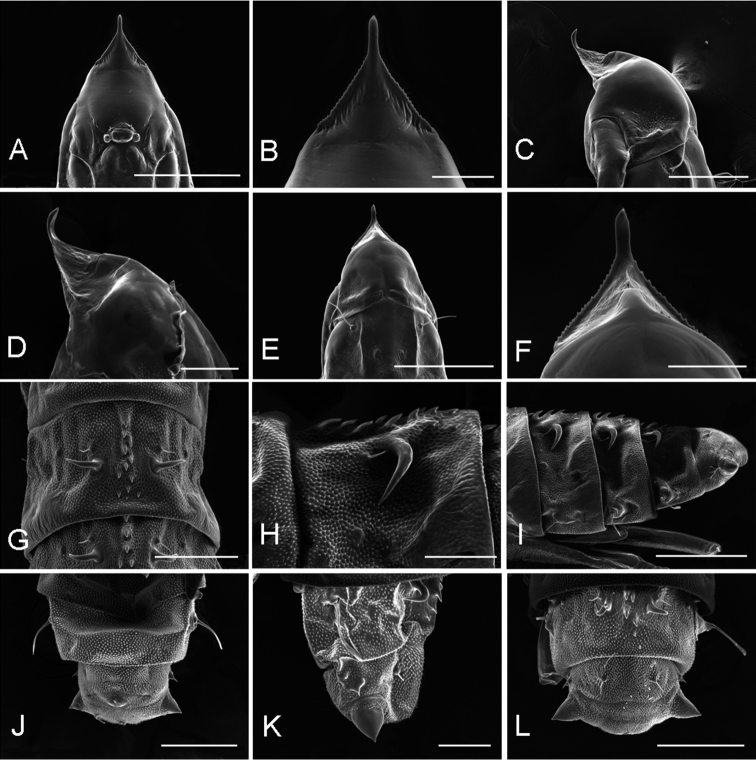
Pupa of *Phyllocnistis indistincta* from *Cornus controvera*, Japan (Honshu, Nara Prefecture) [Suppl. material 1, No. 78]. **A** head, ventral view **B** cocoon cutter, ventral view **C** head, lateral view **D** cocoon cutter, lateral view **E** head, dorsal view **F** cocoon cutter, dorsal view **G** spines and a pair of setae of abdominal tergum **H** same, lateral view **I** A7–A10, lateral view **J** A8–A10, ventral view **K** same, lateral view **L** same, dorsal view. Scale bars: 400 µm **(A**, **E)**, 300 µm **(I)**, 200 µm **(C, G, J, L)**, 100 µm **(B, D, F, H, K)**.

##### Pupa.

Not studied. No description was provided in Ermolaev (1987) and the authors were unable to preserve the pupa from the rearings of the specimens collected in Hokkaido.

##### Biology.

Original description of mine: “Mine is green whitish without frass, more often on the lower side of the leaves of *Cornus controversa*” (Ermolaev 1987). In the present study, the larvae form a serpentine epidermal mine; frass line is pale brown to black. Two mines were found on the upper side of the leaf on *C. controversa*. The final instar larva spun a white cocoon at the leaf margin, the leaf margin slightly curled upwards by contraction of the cocoon silk.

##### Phenology.

No data were provided in the original description of Ermolaev (1987). In Japan (Hokkaido), the pupa was found in late October; there are likely two generations annually. The overwintering form of this species is unknown.

##### Ecology and host plants.

The host plant is *Cornus controversa* Hemsl. (Ermolaev 1987). In Japan, Hokkaido, the species has also been found on this plant.

##### Distribution.

Russia: Kunashir island, Alekhino and Tretyakovo (Ermolaev 1897); Japan: Hokkaido, Sorachi, Kamifurano (present study).

##### Remarks.

In his paper, Ermolaev (1987) indicated that the holotype was stored in the collection of the Zoological Institute of the Russian Academy of Sciences (ZIN RAS), Saint Petersburg, Russia. The curator at the time, who also collaborated with Ermolaev, Dr V.I. Kuznetzov, has unfortunately passed away. The current curator, Dr S.V. Baryshnikova (Seksyaeva) informed the authors that the holotype of *P. cornella* is not in the collection of ZIN RAS and has never been deposited there. As Ermolaev worked in Vladivostok, the authors tried to locate the specimens in the collections at the Biological and Soil Institute of the Far Eastern Branch of the Russian Academy of Sciences (present: Federal Scientific Center of the East Asia Terrestrial Biodiversity FEB RAS), but Dr M.G. Ponomarenko kindly informed the authors that there are no any specimens of *P. cornella* in the Institute's collection. Ermolaev was mostly active in the 1970s and 1980s and left science almost 30 years ago, together with all his collections (including *P. cornella*) and nobody knows if he is still living or where he might be.

In Figure 1 in the original description, Ermolaev (1987) draws three apical ciliary strigulae, but, in the text, he writes that there are four, which the authors find more likely to be corrrect. It is believed that the two female specimens that were sampled in Japan, Hokkaido correspond to *P. cornella*. Hokkaido is geographically close to Kunashir and has a similar flora and fauna and the specimens closely fit the original description of the species (Ermolaev 1987). As only two females were sampled and the type locality has not been explored, the neotype selection has been postponed until more material has been collected on Hokkaido and Kunashir Islands.

## Discussion

Overall, 17 species of *Phyllocnistis* are presently known in the Asian part of Russia, China and Japan (Table 1). Amongst them, so far only one *Phyllocnistis* species, *P. cornella*, has been known to develop on plants of the family Cornaceae (Table 1). Here the authors confirm the existence of three other Cornaceae-feeding *Phyllocnistis*: *P. indistincta* (Japan), *P. saepta* (China) and *P. verae* (Russia), which were discovered during a DNA barcoding campaign for leaf-mining insects of Northeast Asia. The average interspecific divergence for the DNA barcode fragment found within the complex of *Phyllocnistis* spp. feeding on *Cornus* reaches 13.8%, which is comparable to other members of the Phyllocnistinae (Table 2). The divergent split in COI was supported by the two sequenced nuclear genes H3 and 28S (except that, for *P. indistincta* and *P. saepta*, no difference was found in 28S). In addition to the high genetic divergence, morphological differences were found in wing pattern, male and female genitalia, allowing the authors to describe the three new species developing on *Cornus* in Northeast Asia and distinguish them from the previously known *P. cornella*.

**Table 1.** *Phyllocnistis* species occurring in the Asian part of Russia, China and Japan, and their host plants.

No.	*Phyllocnistis* species^1^	Host plant family and species	Distribution	Reference
5	*P. chlorantica* Seksyaeva, 1992	Chloranthaceae: *Chloranthus japonicus*	Russia: Russian Far East (Primorskiy Kray); Japan (Hokkaido, Honshu, Kyushu)	Kobayashi et al. 2011, Seksyaeva 1992, Sinev 2008
6	*P. shizukagozen* Kobayashi & Hirowatari, 2011	Chloranthaceae: *Chloranthus serratus*, *Sarcandra glabra*	Japan (Honshu, Kyushu)	Kobayashi et al. 2011
1	*P. cornella* Ermolaev, 1987	Cornaceae: *Cornus controversa*	Russia: Russian Far East (Kunashir); Japan (Hokkaido)	Ermolaev 1987,present paper
2	*P. indistincta* Kobayashi & Triberti sp. n.	Cornaceae: *Cornus controversa, C. florida, C. macrophylla, C. kousa*	Japan (Honshu, Shikoku, Kyushu)	present paper
4	*P. saepta* Kirichenko, Ohshima & Huang sp. n.	Cornaceae: *Cornus macrophylla*	China (Yunnan)	present paper
3	*P. verae* Kirichenko, Triberti & Lopez-Vaamonde sp. n.	Cornaceae: *Cornus alba*	Russia: Siberia (Krasnoyarsk Kray)	present paper
7	*P. hyperbolacma* (Meyrick, 1931)	Daphniphyllaceae: Daphniphyllum macropodum subsp. humile	Japan (Honshu)	Meyrick 1931, Kuroko 1982
8	*P. selenopa* Meyrick, 1915	Meliaceae: *Melia azedarach*	Japan (Honshu, Shikoku, Kyushu)	Kuroko 1982, Kobayashi and Hirowatari 2013
13	*P. embeliella* Liu & Zeng, 1989	Myrsinaceae: *Embelia lacta*	China (Guangdong)	Liu and Zeng 1989
9	*P. citrella* Stainton, 1856	Rutaceae:*Citrus* spp., *Poncirus trifoliata*, *Fortunella* spp.	Japan (Honshu, Shikoku, Kyushu, Ogasawara), China (Jiangsu, Zhejiang, Fujian, Hubei, Hunan, Guangdong, Hainan, Chongqing, Guizhou, Guangxi, Sichuan, Yunnan)	Kuroko 1982, Kobayashi and Hirowatari 2013
14	*P. wampella* Liu & Zeng, 1985	Rutaceae: *Clausena lansium*	China (Guangdong)	Liu and Zeng 1985
10	*P. gracilistylella* Kobayashi & Jinbo, Hirowatari, 2011	Salicaceae: *Salix gilgiana*, *S. gracilistyla*, *S. integra*, *S. serissaefolia*	Japan (Honshu, Kyushu)	Kobayashi et al. 2011
11	*P. labyrinthella* (Bjerkander, 1790)	Salicaceae: *Populus alba*, *P. tremula* (Siberia, Russian Far East), *P. nigra* (Siberia)	Russia: Siberia (Novosibirsk, Kemerovo, Irkutsk Oblasts, Krasnoyarsk Kray, Republic of Altai, Republic of Yakutia); Russian Far East (Khabarovsk Kray, Primorskiy Kray, Kuril Islands)	Ermolaev 1987, Seksyaeva 1997, Kuznetzov and Baryshnikova 2004, Sinev 2008
12	*P. saligna* (Zeller, 1839)	Salicaceae: *Salix fragilis*, *S. kochiana* (Siberia); *Salix* sp. (Russian Far East); *S. babylonica*, *S. bakko*, *S. chaenomeloides*, *S. gilgiana*, *S. gracilistyla*, *S. integra*, *S. miyabeana*, *S. reinii*, *S. sachalinensis*, *S. serissaefolia* (Japan)	Russia: Siberia (Novosibirsk, Kemerovo Oblasts); Russian Far East (Primorskiy Kray); Japan (Hokkaido, Honshu, Kyushu, Shikoku), China (Heilongjiang, Jilin, Liaolin, Hebei, Shanxi)	Martynova 1955, Barannik 1981, Kagata and Ohgushi 2001, Kobayashi et al. 2011
15	*P. unipunctella* (Stephens, 1834)	Salicaceae: *Populus nigra*, *P. balsamifera* (Siberia); *P. nigra*, *P. suaveolens* (Russian Far East); P. nigra var. italica (Japan)	Russia: Siberia (Novosibirsk, Irkutsk Oblasts, Republic of Yakutia), Russian Far East (Primorskiy Kray, Kuril Islands), Japan (Hokkaido, Honshu),	Tomilova 1973, Ermolaev 1987, Sinev 2008, Kobayashi et al. 2011
16	*P. toparcha* Meyrick, 1918	Vitaceae: *Vitis* spp., V. ficifolia var. lobata, *V. flexuosa*, Ampelopsis glandulosa var. heterophylla, *Parthenocissus tricuspidata*, *Cayratia japonica*	Japan (Hokkaido, Honshu, Shikoku, Kyushu, Ryukyu)	Meyrick 1931, Kuroko 1982
17	*P. vitella* Ermolaev, 1987	Vitaceae: *Vitis amurensis*	Russia: Russian Far East (Primorskiy Kray)	Ermolaev 1987, Sinev 2008
**Further candidate species**				
18	*Phyllocnistis* sp. 1	Salicaceae: *Salix* sp.	Russia: Russian Far East (Primorskiy Kray)	Col: N. Kirichenko
19	*Phyllocnistis* sp. 2	Vitaceae: *Parthenocissus tricuspidata*	Japan (Honshu)	Col: E.J. van Nieukerken
20	*Phyllocnistis* sp. 3	Fabaceae: *Derris trifoliata*	Japan (Okinawa)	Col: A. Kawakita
21	*Phyllocnistis* sp. 4	Oleaceae: *Ligustrum japonicum*	Japan (Shikoku)	Col: A. Kawakita

**^1^** listed according to the host plant family.

Endosymbiotic bacteria like *Wolbachia* may manipulate host reproduction and significantly affect mitochondrial divergence within insect species (Kodandaramaiah et al. 2013, Ritter et al. 2013). In this case, deep mitochondrial splits observed in *Cornus*-feeding *Phyllocnistis* species were not associated with *Wolbachia* infections, since amongst the 14 specimens of the four *Phyllocnistis* species, only one specimen (i.e. *P. indistincta*, MICRU069-16; Honshu, Japan) showed evidence of *Wolbachia* infection (Suppl. material 2). Such mitochondrial divergences could be the result of geographic isolation. Thus, *P. cornella* is known only from the neighbouring islands – Kunashir (Russia) and Hokkaido (Japan), whereas *P. indistincta* was found on Honshu, Shikoku and Kyushu (Japan), *P. saepta* in Yunnan Province (China) and *P. verae* in Central Siberia (Russia). Despite an extensive survey in 2015–2017, *P. verae* was not found in Western or Eastern Siberia, nor in the Russian Far East.

*Phyllocnistis* species develop on different *Cornus* species: *P. indistincta* on *C. controversa, C. kousa, C. macrophylla* (native to Japan) and on *C. florida* (introduced in Japan from North America), *P. saepta* on *C. macrophylla* (native to China) and *P. verae* on *C. alba* (native to Siberia). The related species *P. cornella* was described from the Russian Far East from *Cornus controversa*. The above-mentioned *Cornus* species correspond to four subgenera: *Kraniopsis* (*C. alba* and *C. macrophylla*), *Mesomora* (*C. controversa*), *Syncarpea* (*C. kousa*) and *Cynoxylon* (*C. florida*) (Xiang et al. 2006). The related nature of host plants may partly explain the phylogenetic proximity of some *Cornus*-feeding *Phyllocnistis* species. Amongst four *Phyllocnistis* species, the species from China and Russia are the closest neighbours on the COI tree (minimum intraspecific distance 6.1%) and they both feed on plants from the same subgenus, *Kraniopsis*. Such a relationship is, however, not evident for the other two *Cornus*-feeding *Phyllocnistis* species from Japan.

Dogwoods are widely used for ornamental purposes in gardens and landscaping and their distribution is ubiquitous in temperate Eurasia. The North American *C. florida* is planted in Japan as an ornamental tree. *Cornus alba*, that is native in Russia, Mongolia, Northeast China, Manchuria and North Korea, has been introduced in Europe (Drake 2009). During the course of the study, the authors did not observe dense populations of *Phyllocnistis* on *Cornus* in the surveyed locations and, therefore, it is unclear whether or not they may impact their hosts in urban ecosystems. Abundant populations of another leaf mining moth, *Antispila sinensis* Liu & Wang, 2017 (Heliozelidae), recently described from *Cornus walteri* Wangerin in China, noticeably damaged the foliage of shade trees in Chinese city parks (Liu and Wang 2017).

In addition to the three new species of *Phyllocnistis* discovered on *Cornus* in Northeast Asia, the analysis of DNA barcodes allowed the detection of a further four putative new species of *Phyllocnistis* in this region, though on plant families other than Cornaceae (Table 1, Suppl. material 1). One species (mentioned in the text as *Phyllocnistis* sp. 1) was found in the Russian Far East, Primorskiy Kray on *Salix* (Salicaceae) (N. Kirichenko leg.), while the other three were sampled as single individuals in Japan: *Phyllocnistis* sp. 2 on *Parthenocissus* (Vitaceae) (E.J. van Nieukerken leg.), *Phyllocnistis* sp. 3 on *Derris* (Fabaceae) and *Phyllocnistis* sp. 4 on *Ligustrum* (Oleaceae) (A. Kawakita leg.). The putative new species on *Ligustrum* represents the first record of *Phyllocnistis* feeding on Oleaceae (Table 1). Additional specimens will be needed for the formal description of these species.

## Supplementary Material

XML Treatment for Phyllocnistis indistincta

XML Treatment for Phyllocnistis verae

XML Treatment for Phyllocnistis saepta

XML Treatment for Phyllocnistis cornella

## Supplementary materials

Supplementary material 1 Table S1. *Phyllocnistis* species involved in the study. Data type: specimens data Explanation note: Where pertinent, genitalia preparation number and sex are given in square brackets in the Sample ID column. Both the Process ID and Sample ID codes link the record in the BOLD database and the voucher specimen from which the sequence is derived. This is an open access article distributed under the terms of the Creative Commons Attribution License (CC BY 4.0), which permits unrestricted use, distribution, and reproduction in any medium, provided the original author and source are credited.Natalia Kirichenko, Paolo Triberti, Shigeki Kobayashi, Toshiya Hirowatari, Camiel Doorenweerd, Issei Ohshima, Guo-Hua Huang, Min Wang, Emmanuelle Magnoux, Carlos Lopez-Vaamonde

Supplementary material 2 Figure S1. The DNA barcoded specimens of Cornus-feeding *Phyllconistis* tested for presence of Wolbachia and other Rickettsiaceae Data type: molecular data Explanation note: The serial number of the specimens (№ 1-14) on the COI tree (**A**) correspond to those on the images of agarose gels (**B**). The positive control is indicated by “+”, negative by “H2O”. The infected *Phyllocnistis* specimen MICRU069-16 from Japan (Honshu) is shown under № 3 and additionally shaded in red on the COI tree. (Tests on presence of Wolbachia and other Rickettsiaceae) This is an open access article distributed under the terms of the Creative Commons Attribution License (CC BY 4.0), which permits unrestricted use, distribution, and reproduction in any medium, provided the original author and source are credited.Natalia Kirichenko, Paolo Triberti, Shigeki Kobayashi, Toshiya Hirowatari, Camiel Doorenweerd, Issei Ohshima, Guo-Hua Huang, Min Wang, Emmanuelle Magnoux, Carlos Lopez-Vaamonde
