# Supplementary material for: Systematics of Phyllocnistis leaf-mining moths (Lepidoptera, Gracillariidae) feeding on dogwood (Cornus spp.) in Northeast Asia, with the description of three new species
Source: Zookeys. 2018 Feb 8;(736):79–118. doi: 10.3897/zookeys.736.20739 (PMC5906744; doi:10.3897/zookeys.736.20739)
Supplement: Supplementary material 2 — Figure S1. The DNA barcoded specimens of Cornus-feeding Phyllconistis tested for presence of Wolbachia and other Rickettsiaceae [file zookeys-736-079-s002.pdf]

ZooKeys

Systematics of *Phyllocnistis* leaf-mining moths (Lepidoptera: Gracillariidae) feeding on dogwood (*Cornus* spp.) in Northeast Asia, with the description of three new species

Natalia Kirichenko[1,2], Paolo Triberti[3], Shigeki Kobayashi[4], Toshiya Hirowatari[5], Camiel Doorenweerd[6,7], Issei Ohshima[8], Guo-Hua Huang[9], Min Wang[10], Emmanuelle Magnoux[11], Carlos Lopez-Vaamonde[11,12]

[1]Sukachev Institute of Forest SB RAS, Akademgorodok 50/28, 660036, Krasnoyarsk, Russia; nkirichenko@yahoo.com

[2]Siberian Federal University, 79 Svobodny pr., 660041, Krasnoyarsk, Russia

[3]Museo Civico di Storia Naturale, Lungadige Porta Vittoria 9, I37129, Verona, Italy; caloptilia@alice.it

[4]Entomological Laboratory, Graduate School of Life and Environmental Science, Osaka Prefecture University, Sakai, Osaka, 599-8531, Japan; crossroad1994@hotmail.co.jp

[5]Entomological Laboratory, Faculty of Agriculture, Kyushu University, 6-10-1 Hakozaki, Fukuoka, 812-8581 Japan; hirowat_t@agr.kyushu-u.ac.jp

[6]Department of Plant and Environmental Protection Sciences, University of Hawaii, 3050 Maile Way, 96822, Honolulu, United States of America;

[7]Naturalis Biodiversity Center, PO Box 9557, NL-2300 RA Leiden, The Netherlands

[8]Department of Life and Environmental Sciences, Kyoto Prefectural University, Kyoto, Japan, issei@kpu.ac.jp

[9]Hunan Provincial Key Laboratory for Biology and Control of Plant Diseases and Insect Pests, Hunan Agricultural University, Changsha 410128, Hunan, China; ghhuang@hunau.edu.cn

[10]Department of Entomology, South China Agricultural University, Guangzhou 510642, Guangdong, China; minwang@scau.edu.cn

[11]INRA, UR0633 Zoologie Forestière, F-45075 Orléans, France; emmanuelle.magnoux@inra.fr, carlos.lopez-vaamonde@inra.fr

[12]Institut de Recherche sur la Biologie de l'Insecte, CNRS UMR 7261, Université François-Rabelais de Tours, UFR Sciences et Techniques, 37200 Tours, France

Corresponding author: Natalia Kirichenko (nkirichenko@yahoo.com)

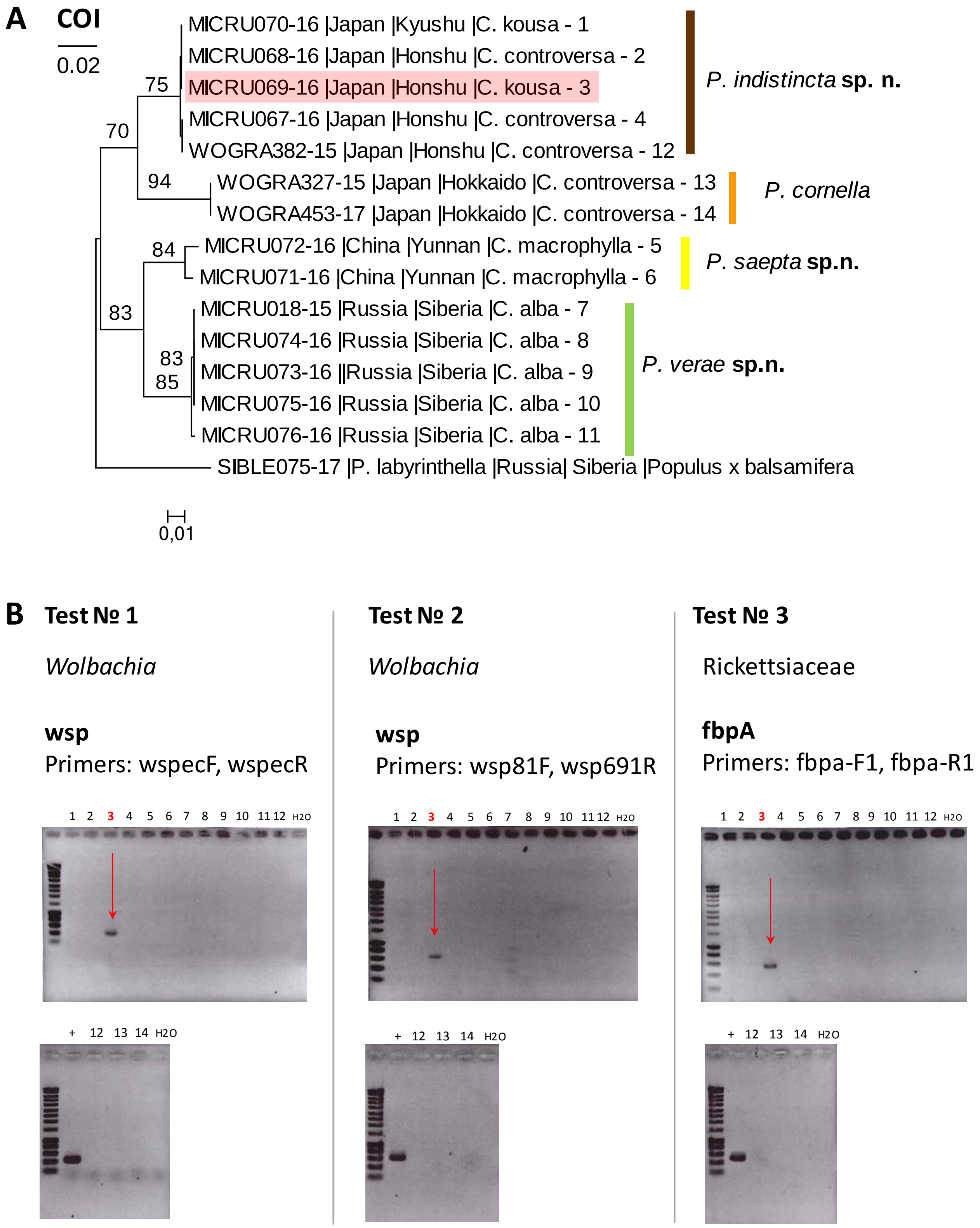

Figure S1. The DNA barcoded specimens of *Cornus*-feeding *Phyllconistis* tested for presence of *Wolbachia* and other Rickettsiaceae. The serial number of the specimens (№ 1-14) on the COI tree (A) correspond to those on the images of agarose gels (B). The positive control is indicated by "+", negative by "H2O". The infected *Phyllocnistis* specimen MICRU069-16 from Japan (Honshu) is shown under № 3 and additionally shaded in red on the COI tree.
